# Paired Expression Analysis of Tumor Cell Surface Antigens

**DOI:** 10.3389/fonc.2017.00173

**Published:** 2017-08-21

**Authors:** Rimas J. Orentas, Sivasish Sindiri, Christine Duris, Xinyu Wen, Jianbin He, Jun S. Wei, Jason Jarzembowski, Javed Khan

**Affiliations:** ^1^Lentigen Technology, Inc., a Miltenyi Biotec Company, Gaithersburg, MD, United States; ^2^Genetics Branch, National Cancer Institute, Center for Cancer Research, National Institutes of Health, Bethesda, MD, United States; ^3^Department of Pathology, Medical College of Wisconsin, Milwaukee, WI, United States

**Keywords:** neuroblastoma, gene expression profiling, chimeric antigen receptor-T cells, immunotherapy, GFRA3, GFRA2, GPR173, pediatric oncology

## Abstract

Adoptive immunotherapy with antibody-based therapy or with T cells transduced to express chimeric antigen receptors (CARs) is useful to the extent that the cell surface membrane protein being targeted is not expressed on normal tissues. The most successful CAR-based (anti-CD19) or antibody-based therapy (anti-CD20) in hematologic malignancies has the side effect of eliminating the normal B cell compartment. Targeting solid tumors may not provide a similar expendable marker. Beyond antibody to Her2/NEU and EGFR, very few antibody-based and no CAR-based therapies have seen broad clinical application for solid tumors. To expand the way in which the surfaceome of solid tumors can be analyzed, we created an algorithm that defines the pairwise relative overexpression of surface antigens. This enables the development of specific immunotherapies that require the expression of two discrete antigens on the surface of the tumor target. This dyad analysis was facilitated by employing the Hotelling’s *T*-squared test (Hotelling–Lawley multivariate analysis of variance) for two independent variables in comparison to a third constant entity (i.e., gene expression levels in normal tissues). We also present a unique consensus scoring mechanism for identifying transcripts that encode cell surface proteins. The unique application of our bioinformatics processing pipeline and statistical tools allowed us to compare the expression of two membrane protein targets as a pair, and to propose a new strategy based on implementing immunotherapies that require both antigens to be expressed on the tumor cell surface to trigger therapeutic effector mechanisms. Specifically, we found that, for MYCN amplified neuroblastoma, pairwise expression of ACVR2B or anaplastic lymphoma kinase (ALK) with GFRA3, GFRA2, Cadherin 24, or with one another provided the strongest hits. For MYCN, non-amplified stage 4 neuroblastoma, neurotrophic tyrosine kinase 1, or ALK paired with GFRA2, GFRA3, SSK1, GPR173, or with one another provided the most promising paired-hits. We propose that targeting these markers together would increase the specificity and thereby the safety of CAR-based therapy for neuroblastoma.

## Introduction

The field of immunotherapy has entered a time of rapid advancement. Built upon decades of basic research, the fields of recombinant protein engineering, high-throughput screening, and gene vector biology, have allowed the implementation of engineered immunoglobulin molecules and engineered immune cells in clinically meaningful protocols. This is especially true for hematologic malignancies. Antibodies featuring engineered Fc domains or bispecific antibodies are now considered part of our current armamentarium for leukemia expressing CD19 or CD20 (1–3); as are T cells engineered to express chimeric antigen receptors (CARs) that target CD19 (4, 5). The targeting of B cell malignancies by these agents also eliminates normal mature B lymphocytes, which is a well-tolerated “on-target” side-effect. Moreover, the ability to use these approaches in conjunction with hematopoietic stem cell transplantation can take patients who are complete responders, and who may yet relapse, into the realm of true “cure” (6).

The ability to classify tumors according to their gene expression profile has resulted in an explosion of useful approaches to differentiate among tumor types, to clarify pathological anomalies, and to create meaningful sub classifications of disease that have informed further investigation. In 2000, Alizadeh et al. used DNA microarrays to classify diffuse large B-cell lymphoma into two new types, germinal center-like and activated B-cell like (7). More than a decade later, this basic principle was used to redefine how we classify medulloblastoma (8). In 2007, Wood et al. published a landmark study in which the entire transcriptome of 11 breast and 11 colorectal tumors was compared to the Reference Sequence database, thus analyzing 20,857 transcripts from 18,191 genes (9). This study identified the key conserved mutations across two specific tumor types in reference to normal transcriptomes. The need to have an even more robust definition of a “normal” transcript dataset led us to generate a database of mRNA gene expression profiles from 158 human samples (19 different organs from 30 different donors) for 18,927 unique genes (10). During the creation of this database, we demonstrated its ability to identify 19 neuroblastoma tumor-specific genes, in comparison to normal. The data for this, now expanded, set of normal and pediatric tumor gene expression analysis can be found online, hosted by the NCI, at: http://home.ccr.cancer.gov/oncology/oncogenomics/ in the “data” section. Given the ability to now rapidly analyze whole transcriptomes, our attention turned to the ability to identify new tumor-expressed targets for antibody-based or CAR-based therapy.

Using whole transcriptome-based analyses filtered for plasma membrane protein expression generates a data set referred to as the “surfaceome.” However, there is a key methodological gap remaining in this approach. There is no single database or filter that definitively identifies transcripts encoding plasma membrane proteins. Biochemical methods, wherein surface structures are chemically labeled or tagged, isolated, and then subjected to mass spectrometry are still under development, and are incomplete with respect to the catalog of surface targets identified (11, 12). Here, we present our method of consensus scoring, wherein data from multiple databases are aggregated, and a plasma membrane residence score assigned to each transcript. In our previous work, we averaged the gene expression level of each transcript across 12 types of pediatric cancers and compared each to an average collection of normal tissues (13). Here, we have updated this approach by using RNASeq data. More importantly, we have now developed a new analytical tool that allows overexpressed transcript to be scored as a dyad, that is, the two proteins that are most overexpressed in cancer versus normal tissue as a pair can now be identified.

The ability to target hematologic malignancies with immunotherapeutic agents is becoming a well-established approach. However, progress against “solid cancers” has been slower, due to at least two factors. The first is the inherent cellular complexity of the tumor lesion itself. Second, especially in reference to pediatric malignancies that have the lowest mutation rates of all cancers (14), there may be no single target that can sufficiently define a cellular target as being “cancer” or “normal.” For example, in our previous gene expression profiling work, the top 3 transcripts overexpressed in stage 4 neuroblastoma were SLC10A4, CHRNA3, and SLC29A4 (13). These encode for a Na^+^/bile cotransporter-like protein in the solute carrier superfamily (SLC), a subunit of the nicotinic cholinergic receptor, and an SLC family member involved in nucleoside transport, respectively. None of these hits appear safe to target, as they are likely to be broadly expressed in normal or essential tissues, even though gene expression profiling informs us that they are highly expressed in neuroblastoma as compared to normal tissue. In this report, we present a new approach, using neuroblastoma as our example, for identifying pairs of ligands that might be more safely targeted than any single target, giving another dimension of target specificity to our anticancer therapeutic approach. This approach can be similarly applied to any RNASeq data set derived from other malignancies. An important limitation to these approaches is our inability to define the “glycome” of solid tumors in a high throughput manner (15). This is an important consideration as the most promising CAR and antibody-based approaches to treat neuroblastoma to date, focus on the ganglioside GD2 (16, 17). As GD2 and other glycoform-based approaches are being refined, we propose that our current approach holds promise for developing “two-hit” gated approaches for solid tumor immunotherapy.

## Materials and Methods

### Neuroblastoma and Normal Tissues Sources for RNASeq

PolyA selected RNA libraries of neuroblastoma (NB) samples were prepared for RNA sequencing on an Illumina HiSeq2000 using the manufacturer’s protocol (Illumina, Inc., San Diego, CA, USA). A total of 32 stage 4 *MYCN*-amplified (*MYCN*-A) and 70 *MYCN*-non-amplified (*MYCN*-NA) neuroblastomas were analyzed. For normal tissue, 17 samples from brain and 46 from other tissues were analyzed. Raw sequencing files were converted to FASTQ format and were mapped to the human reference genome (GRch37) using Tophat2.2. Using PICARD and Samtools, a QC check was performed on the produced BAM files and PCR duplicate reads were removed.

### Building a Cell Surface Annotation File

In total, 6,414 transcripts (as annotated in RefSeq) from the human genome were chosen for downstream analysis. This was accomplished by gathering information available for each transcript from the following annotation or aggregation databases:

(1) from Compendia
  (a) isSurfaceomeSurfaceProtein (see Ludwig Institute for Cancer Research, Instituto de Bioinformacia e Biotecnologia, Brazil, http://www.bioinformatics-brazil.org/surfaceome/home)
  (b) isCDDSurfaceProtien, Conserved Domain Database (see http://www.ncbi.nlm.nih.gov/cdd)
  (c) isGOSurfaceProtein, Gene Ontology Consortium (see http://geneontology.org)
  (d) isSurfaceProteinEvidence
(2) *from the Pandey Lab*, Johns Hopkins University (http://pandeylab.igm.jhmi.edu)
  (e) AmiGo (GO) (see http://amigo.geneontology.org/amigo)
(3) *Compartments, from the Jensen Lab*, Novo Nordisk Foundation for Protein Research, University of Copenhagen (18)
  (f) Knowledge (COMPARTMENTS, based on UniProtKB, MGI, SGD, Flybase, and WormBase, see http://compartments.jensenlab.org/Search)
  (g) Experiments [derived from human protein atlas, see Li et al. (19)] (19)
  (h) Prediction [aggregates WoLF PSORT (20); and YLoc (21, 22)]
  (i) Text mining (text mining of Medline abstracts).

If a transcript was scored as a plasma membrane protein in at least seven of the listed databases, this transcript was considered a positive hit and entered into subsequent analysis. In total, 6,414 cell surface genes were defined for analysis.

### Differential Gene Expression Using Limma/Voom and Tandem Gene Expression Analysis

Linear Models for Microarray and RNA-Seq Data (Limma) is an open-source software package that processes expression array and RNA-Seq data (using the voom function) allowing for differential gene expression analysis (23–25). The two groups analyzed were *MYCN*-A and stage IV *MYCN*-non-amplified (*MYCN*-NA) neuroblastoma. These were compared against normal samples. For a transcript to be considered as overexpressed, a log fold-change (FC) ≥2, expression greater than two FPKM (fragments per kilobase transcript per million mapped reads), and a highly significant *p-*value (*p*val) ≤ 0.001, was required. This analysis identified in 158 for *MYCN*-A neuroblastoma, and 179 for *MYCN*-NA neuroblastoma transcripts.

We then paired each overexpressed cell surface protein-encoding transcript with each of the other transcripts for statistical testing. Hotelling’s *T*-squared test (MANOVA/Hotelling–Lawley multivariate analysis of variance) was used to identify gene pairs, affected together by difference in sample conditions (tumor versus normal tissue). To assess linear dependence between the two genes in a gene pair, the Pearson correlation coefficient (only in tumor) was used. Significant gene pairs (Hotelling’s *p-*value ≤ 0.01) with high correlation value (*r*^2^ *p-*value ≤ 0.05) were chosen as final candidate gene pairs (326 gene pairs comprising 27 unique genes in *MYCN*-A neuroblastoma and 529 gene pairs comprising 34 unique genes in *MYCN*-NA neuroblastoma).

In sum, we applied the following filters to create the final set of gene pairs: (a) MANOVA pval ≤ 0.01, (b) Sort Pearson correlation and pval ≤ 0.05, (c) Median_Exp_gene1 [log_2_(FPKM)] ≥ 2; Median_Exp_gene2 [log_2_(FPKM)] ≥ 2, (d) Log_FC_Gene1 (compared to normal) ≥ 2; Log_FCGene2 (compared to normal) ≥ 2, (e) Log_FC_Gene1 (compared to only brain) ≥ 2; Log_FC_Gene2 (compared to only brain) ≥ 2, (*f*) Log FKPM in any vital organ was varied between ≤1.0, 1.5, and 2.0.

### Pathological Analysis

Antibodies specific for cell surface antigens were obtained from the following sources and used on an automated Leica Bond staining platform as indicated at the Department of Pathology, Medical College of Wisconsin: anaplastic lymphoma kinase (ALK)-1 (Leica NCL-L-ALK, clone 5A4, 1:200 dilution, control staining on a normal tissue blocks including cerebellum, pancreas, tonsil, and lymphoma/ALL was carried with all antibodies listed unless otherwise noted, positive staining for ALK on ALL noted), Cadherin 24 (CDH24) (LC Bio LS-C168610 pAB rabbit, 1:100 dilution, positive control block and ganglion cells/nerve cells of bowel wall, endometrium surface epithelium, and hepatocellular carcinoma were also stained with positive staining for each of these three additional specimens), DLK (AbCam ab21692 pAB rabbit, 1:300 dilution, control cell block was stained and positive signal was noted additionally for placenta, with islets and ductal cells staining positive, and neutrophils staining positive), GFRA2 (Sigma HPA 024701 pAB rabbit, 1:20 dilution, control cell block and additional tissues including macrophages and sinusoids in liver, tonsil leukocytes, which were positive and ALL, weakly positive, and lung, unremarkable), GFRA3 (Sigma HPA 020731 pAB rabbit, 1:500 dilution, control cell block and additional tissue including increased staining in pancreatic ductal epithelium and islets, decreased in exocrine glandular cells, strong lymphatic and positive alveolar macrophage staining), GPR173 (Novus BioNLS51 pAB rabbit, 1:300 dilution, control block and additional stain for red cells, positive, lymphocytes, and epidermis), TrkA (Abcam ab76291, 1:100 dilution, control block and tonsil, reticular dendritic network positive, liver (negative except for arteries), positive pancreas islet cells, sinusoids of HCC, and some positivity in hepatocytes).

Tissue microarrays containing neuroblastoma were purchased from US Biomax (Derwood, MD, USA), specifically a microarray panel with neuroblastoma and peripheral nerve tissue, 32 cases (27 neuroblastoma and 5 normal peripheral nerve tissue)/64 cores (MC642), derived from retroperitoneal (38 cores), pelvic cavity (2 cores), mediastinum (6 cores), and adrenal (8 cores) disease sites were stained for each antibody. Normal and neuroblastoma cores were scored 0, 1, 2, 3 according to staining intensity. Images were processed on NDP.view 2 software (Hamamatsu Photonics, Inc.), and magnified, as indicated, for presentation. Normal controls to confirm antibody staining were from de-identified surplus tissue from the hospital core lab. All tissues were used in conformity to established ethical policies. Commercially obtained tissue (US Biomax, Derwood, MD, USA) was obtained under HIPPA approved protocols. For RNASeq, informed consent from each participant or guardian was obtained by qualified investigators at local Children’s Oncology Group institutes or at the NCI and samples were collected after approval by the local Institution Review Boards. All samples were anonymized and the study deemed exempt by the Office of Human Subject Research, NIH.

## Results

**Figure 1:**
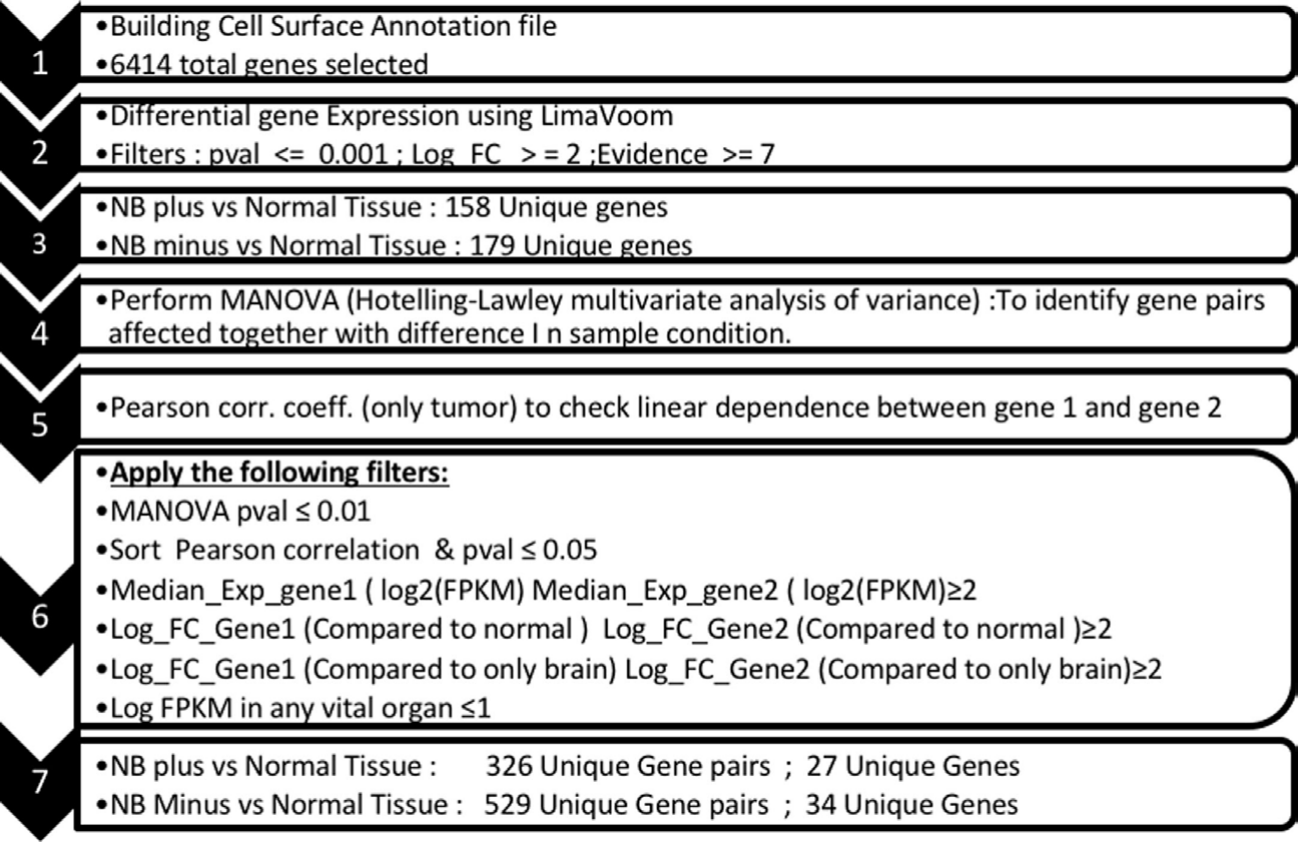
Bioinformatic pipeline for identifying overexpressed gene pairs. Identification of tandem pairs of overexpressed genes (versus normal) began with building a file of transcripts known to be expressed in our cancers of interest, being advanced stage neuroblastoma (NB) with (plus) or without (minus) MYCN gene amplification (1), followed by scoring the individual genes represented in the cell surface transcriptome database, using 9 databases or filters (here, we required a positive hit in at least seven of the sources and a greater than twofold expression of the cancer-associated transcript over normal) (2), these hits were then analyzed by Hotelling–Lawley (4) to identify gene pairs expressed together, a Pearson co-efficient of tumor only to check for dependence of the transcripts in the tumor (5), and then filter sets applied (6). These variables are adjustable in the online database and allow thresholds to be set for gene expression (FPKM medians), expression of the gene pairs versus normal tissues, versus brain, and versus a subset of vital organs (liver, heart, lung, kidney), and gave a final readout of 26 unique genes for NB plus and 33 unique genes for NB minus (7). See Orentas et al. (13, 26).

Using the data analysis pipeline described in Section “Materials and Methods,” and illustrated in Figure 1, we set the final parameter of Log FKPM in any vital organ at 1.0, and received a readout of 325 unique gene pairs; comprised of 26 unique genes for *MYCN*-A neuroblastoma and 528 unique gene pairs, comprised of 33 unique genes, for *MYCN*-NA neuroblastoma. In Table 1, we present the top scoring genes that comprised the pairs we identified for both disease types as a single list. We were surprised by the brevity of the list and experimented with loosening the stringency for normal gene expression. Our analysis pipeline first filters for FPKM ≤ 2 for our composite set of normal tissues and our composite set of brain transcripts. We then require FPKM ≤ 1 for a subset of vital organs (heart, liver, lung, kidney). From our previous published work, we know that choice of the normal tissue set used for differential gene expression scoring has a profound impact on analysis outcome (26). When we loosened the vital organ stringency to FPKM ≤ 2, three more genes arose as candidates including DLK1, classified as a “Good” target (Table 1). Using this approach, investigators can set different stringency limits for normal tissues, and tumor-derived expression data sets re-filtered. One can also alternate between different sets of normal antigen expression profiles depending upon specific tissues that may be of concern.

**Table 1:** Individual analysis of over-expressed genes scored for pairwise expression.

Gene symbol	Target suitability	Amplified MYCN±	Note
ACVR2B	Good	(+)	
Anaplastic lymphoma kinase	Good	Both	
Cadherin 24	Good	Both	
CELSR3	Good	(+)	
DLK1	Good	Both	Change FPKM ≤ 2
GFRA2	Good	Both	
GFRA3	Good	Both	
GPR173	Good	Both	
Insulin receptor-related receptor	Good	(−)	
Melanocortin 1 receptor	Good	(+)	
Neurotrophic tyrosine kinase 1	Good	(−)	
PCDHB6	Good	(−)	
PTPRH	Good	(−)	
SDK1	Good	(−)	

CLSTN2	Fair	(−)	
Endothelin converting enzyme like-1	Fair	(−)	
Lysophosphatidic acid receptor 2	Fair	Both	Change FPKM ≤2
NKAIN1	Fair	Both	
SLC10A4	Fair	Both	
SLC29A4	Fair	Both	
TMEM169	Fair	Both	

ADAMTS7	Poor	(−)	Change FPKM ≤2
CHRNA3	ntr	Both	ntr = neurotransmitter receptor
CHRNA5	ntr	Both	
CHRNA7	ntr	Both	
CHRNB4	ntr	Both	
GRM8	ntr	Both	
KCNH4	ntr	Both	
P2RX3	ntr	Both	
SCN9A	ntr	Both	
SLC18A1	ntr	(−)	
SLC18A3	ntr	(−)	
SLC6A2	ntr	Both	

FAM57B	n/a	Both	n/a = intracellular or pseudogene
KIAA1524	n/a	Both	
NRM.3	n/a	Both	
RPRM	n/a	Both	
SLC26A10	n/a	Both	
SORCS1	n/a	(−)	

*After scoring the surface of advanced stage neuroblastoma, both MYCN amplified (+) or non-amplified (−), a small set of genes were found to score highly for tumor and sufficiently low for the filters set for excluding normal tissue. For three of the transcripts, we lowered this threshold for the set of vital organs to FPKM ≤2 (noted above). We segregated gene hits according to their suitability as immunotherapy targets. The hits that were intracellular proteins of pseudogenes were scored as n/a, poor hits were present on vascular tissue (ADAMTS7) or serve as neurotransmitter receptors. Hits classified as good were those with sufficient annotation or published studies confirmed their status as targetable cell surface proteins. Hits classified as Fair had minimal information available*.

In examining the individual genes that comprise our gene pairs, Table 1, it is apparent that our data analysis pipeline can be further refined. Six genes were classified as “n/a” meaning that we will not consider them as targets as they are either pseudogenes or intracellular transcripts [FAM57B, KIAA1524, NRM, RPRM, SLC26A10, SORCS1 (27, 28–30, 31)]. As our pipeline still yielded these hits, we can be sure that there is more work to do in improving the annotation present in current databases and in our ability to filter them for non-plasma membrane expressed transcripts.

### Targets with High Risk Profiles

Transcripts encoding genes in the “Poor” category included ADAMTS7 (32), and a series of neurotransmitter receptor proteins. Cholinergic receptors (CHRNA3, CHRNA5, CHRNA7, CHRNB4) are particularly risky. Our assumption is that they would generate severe systemic toxicity, perhaps recreating myasthenia gravis-like symptoms. Both GRM8 (glutamate receptor, metabotropic 8) and KCNH4 (potassium channel), voltage-gated subfamily H, member 4 (brain restricted) are CNS neurotransmitters. SCN9A (sodium voltage-gated channel alpha subunit 9) and P2RX3 (purogenic receptor P2X, ligand-gated ion channel 3) are involved in nociception signaling. SLC18A1 and SLC18A3 are vesicular monoamine and vesicular acetylcholine transporters, respectively. SLC6A2 is responsible for re-uptake of norepinephrine in presynaptic nerve terminals and is thus also too risky.

### Targets with Moderate Risk Profiles

The group described as “Fair” targets in Table 1 each have some risk. CLSTN2 (calsyntenin 2, CDHR13, cadherin-related 13) is located in postsynaptic membrane of excitatory, primarily GABA-ergic, CNS synapses, although it appears to be developmentally regulated (33, 34). Endothelin converting enzyme like-1 is developmentally important for joint formation and innervation in humans (35). Although it is associated with the cell surface, it is also present in the endoplasmic reticulum, and its expression pattern (neuron-specific) is of concern (36). Lysophosphatidic acid receptor 2 is a G-coupled protein receptor (GPCR) that has been associated with both cancer and lung fibrosis, yet, as a class, these molecules are difficult to target and may be expressed on some normal lung endothelial cells (37, 38). NKAIN1 (Na+/K+ transporting ATPase interacting 1) has been proposed as a prostate cancer marker, but also has some restricted neuronal expression (39, 40). SLC29A4 is a plasma membrane monoamine transporter that functions a serotonin uptake transporter and, therefore, is likely associated with neuronal signaling (41). SLC10A4 is in the Na+/bile acid co-transporter family, little is known of its function, and some CNS expression by immunohisotchemical analysis has been demonstrated (42, 43). TMEM169 has little information associated with it, and thus risk cannot be evaluated.

### Targets with Favorable Risk Profiles

Transcripts encoding 14 plasma membrane-associated proteins were categorized as being favorable targets for immunotherapy. We consider them here by functional groups. The first group of favorable targets are growth factor receptors known to be overexpressed on either tumors or stem cells of the neuronal and hematopoietic lineage. These are activin A receptor, type IIB (ACVR2B), glial cell-derived neurotropic factor (GDNF) family receptor alpha-2 (GFRA2, which binds neurturin), and GFRA3 (which binds artemin). ACVR2B is a transmembrane serine/threonine kinase signaling molecule in the TGF-beta signaling pathway family, which binds to activin and myostatin (44). ACVR2B is strongly expressed in renal childhood neoplasms and could be readily targeted (45). Both GFRA2 and GFRA3 are cell surface GPI-linked proteins that bind neurotrophins, forming complexes with the RET tyrosine kinase to initiate ligand-dependent signaling. Gfra1, Gfra2, and Gfra3, which signal through the RET tyrosine kinase in the presence of GDNF, neurturin, and artemin, respectively, also play a role in hematopoietic stem cell function, conferring survival signals through the Bcl2 family of proteins (46). Finally, neurotrophic tyrosine kinase 1 (NTRK1, TrkA), is a well-known neuroblastoma antigen that binds nerve growth factor (47), and whose expression has been associated with a number of human cancers, often being discovered as an oncogenic fusion protein (48).

Another favorable hit that may be considered a growth factor receptor is melanocortin 1 receptor (MC1R). However, as this receptor is also a G-coupled receptor that also crosses the membrane seven times, we classify it with the other G-coupled receptor found to be a favorable target, GPR173 (SREB3, super conserved receptor in brain or G-protein coupled receptor 173). M1CR binds alpha-melanin stimulating hormone released by sun-damaged keratinocytes, thus promoting eumelanin production in individuals with a non-mutated MC1R. MC1R also mediates anti-inflammatory properties as well and may promote anti-melanoma immunity (49). Little is known about GPCR173 other than it is expressed at the RNA level in brain and has also been called SREB1 (super conserved receptor in brain, based on low variation between species) (50). By virtue of ligand binding by MC1R and the unique extracellular domains of GPCR173, these both may serve as good targets (51).

Four of the favorable hits are adhesion receptors. CDH24 (type 2) is a cell surface protein expressing five extracellular repeat motifs and has the ability to interact with both beta-catenin, alpha-catenin, and p120 catenin (52). Frameshift mutations have been described for CHD24 in some cancers and may be associated with carcinogenesis (53). CELSR3 (cadherin, EGF LAG seven-pass G-type receptor, 3) is a non-classical cadherin in the flamingo family that does not interact with the catenins. This unique class has seven EGF-like repeats, nine cadherin domains, and two laminin repeats in their extracellular domain and seven transmembrane domains. CELSR3 interacts with molecules that govern cell motility during carcinogenesis through the WNT/planar cell polarity signaling pathway (54). CELSR3 is preferentially upregulated in pancreatic and hepatic cancer stellate cells (55) and has been described to guide axonal migration in the CNS (56). Of concern as a single target is its expression on the amacrine cells of the eye, although in depth studies are limited to zebrafish (57). Protocadherin B6 (PCDHB6) is also a neural adhesion molecule. As the name implies, this molecule family is thought to function in cell adhesion. Interestingly, other PCDH family members were demonstrated to stabilize RET signaling in neuroblastoma, indicating that PCDHs play a role in tumor cell signaling and activation (58). SDK1 (sidekick cell adhesion molecule) is a synaptic cell adhesion molecule in the Ig superfamily. SDK1 has been shown to be overexpressed in asbestos-induced lung adenocarcinoma (59, 60), Sdk1 is also expressed in retinal synaptic sites and may be dangerous for this reason. But for our dual targeting approach may prove to be a good hit.

The final category of favorable targets are transmembrane proteins known to regulate cell signaling, adhesion, or activation. These are ALK, a well-studied neuroblastoma antigen, DLK1 (Delta-like 1 homolog), a transmembrane receptor with multiple EGF repeats that regulates adipogenesis and osteogenesis, protein tyrosine phosphatase, receptor type H (PTPRH) or stomach cancer associated protein tyrosine phosphatase 1 (SAP1), and insulin receptor-related receptor (INSRR). ALK has been the focus of intensive study in neuroblastoma for many years [recently reviewed by Mossé (61)] and is an excellent immunotherapy target. DLK1, a non-canonical notch ligand, has multiple EGF domains and is proposed to govern cell growth and differentiation. Elevated expression of DLK1 was reported in neuroblastoma from patients with poor outcome (62) and induction of DLK1 expression in lung cancer activated both notch-dependent signaling and upregulated matrix metalloproteinase MMP9, which increased cellular invasive potential (63). PTPRH (SAP1) has a single intracellular catalytic domain, multiple extracellular fibronectin-type III repeats, and is known to be overexpressed in human cancer (64). In a recent study, association of SAP1 with CEACAM20 (carcinoembryonic antigen related cell adhesion molecule-20) was found to regulate the inflammatory status of gut epithelial cells by regulating the phosphorylation of CEACAM20 by c-Src, with the subsequent association with *syk* (spleen tyrosine kinase) signaling and induction of IL-8 production (65). INSRR functions as an alkali sensor molecule (66). Upon alterations in pH, extracellular domains rearrange and induce the autophosphorylation of internal kinase domains, thus initiating intracellular signaling. One can speculate that the kinase domain is part of the disease process or that advanced disease selects for an environment in which this gene is upregulated.

All the high-quality hits may confer upon cancer cells activation signals that may in some sense be oncogenic drivers. Many interact with the RET kinase or a pathway associated with RET signaling. The targeting of these growth-promoting proteins through immunotherapy may help prevent tumor escape by denying important growth promoting signals to cells, if escape mutants downregulate their expression.

### Pairwise Associations

Having considered the quality of individual hits, we now interrogate the true power of our analysis, and that is examining pairwise associations of targets on the cancer cell surface. Table 2 lists the pairwise hits for both MYCN-A and *MYCN*-NA neuroblastoma (NB). What becomes immediately evident is that the *MYCN*-NA tumor side of the table has much higher *F* values and correspondingly lower *p* values. The highest *F* value for *MYCN*-A NB does not make the top 20 of our curated list for *MYCN*-NA tumors. This may indicate that without MYCN amplification more total mutations (and thus greater deviation from a normal gene expression profile) are required for *MYCN*-NA tumor to progress to advanced disease. For *MYCN*-amplified tumors, the oncogene supplies a strong internal driver already. In this case, the tumor may either arise more rapidly and have a lesser opportunity to accumulate random mutations, or may simply require less total mutations to progress to advanced disease.

**Table 2:** Pairwise ranking of dual targets.

NB non-A^a^		*F*-value^b^	*p* Value^c^	NB MYCNA^d^		*F*-value	*p* Value
ALK_NTRK1		380	7.0 E−51	ACVR2B_GFRA3		193	2.8 E−30
GFRA3_ALK	1	380	7.2 E−51	GFRA3_ALK	1	142	2.9 E−26
GFRA3_NTRK1		334	3.9 E−48	CDH24_ACVR2B		132	2.4 E−25
NTRK1_GFRA2		333	4.5 E−48	ACVR2B_GFRA2		125	1.4 E−24
SDK1_NTRK1		281	1.5 E−44	ACVR2B_ALK		118	6.7 E−24
GFRA3_GPR173	2	268	1.3 E−43	CDH24_ALK	5	115	1.5 E−23
GFRA2_GFRA3	3	263	3.3 E−43	MC1R_ACVR2B		113	2.0 E−23
ALK_INSRR		262	3.5 E−43	ACVR2B_GPR173		109	6.5 E−23
GPR173_NTRK1		256	1.2 E−42	GFRA3_CELSR3		108	8.2 E−23
PCDHB6_NTRK1		253	2.0 E−42	CELSR3_ACVR2B		107	1.2 E−22
PTPRH_NTRK1		247	5.5 E−42	GFRA3_GPR173	2	97	1.4 E−21
INSRR_GFRA3		243	1.1 E−41	ALK_MC1R		89	1.5 E−20
CDH24_NTRK1		232	1.1 E−40	GFRA2_ALK	4	84	7.2 E−20
GFRA3_PTPRH		230	1.5 E−40	ALK_GPR173	6	82	1.4 E−19
INSRR_NTRK1		227	2.9 E−40	GFRA2_GFRA3	3	82	1.3 E−19
INSRR_GFRA2		221	9.3 E−40	GFRA3_CDH24		82	1.5 E−19
GFRA3_PCDHB6		220	2.7 E−38	CELSR3_ALK		76	8.1 E−19
GFRA2_ALK	4	213	4.5 E−39	GFRA2_CDH24	8	73	2.8 E−18
PTPRH_ALK		211	6.6 E−39	CDH24_CELSR3		70	7.8 E−18
SDK1_ALK		205	2.7 E−38	GPR173_GFRA2	7	63	9.7 E−17
CDH24_GFRA3		199	1.1 E−37	GFRA2_CELSR3		61	2.1 E−16
CDH24_ALK	5	198	1.1 E−37	GPR173_CDH24	9	60	2.3 E−16
GFRA3_SDK1		192	4.1 E−37	MC1R_GFRA3		60	2.6 E−16
ALK_GPR173	6	180	7.5 E−36	CDH24_MC1R		57	8.5 E−16
INSRR_PTPRH		180	8.1 E−36	MC1R_GFRA2		57	8.5 E−16
PCDHB6_ALK		180	7.9 E−36	GPR173_CELSR3		54	3.7 E−15
INSRR_GPR173		171	1.8 E−33	MC1R_CELSR3		51	1.0 E−14
INSRR_PCDHB6		158	1.8 E−33	GPR173_MC1R		50	1.4 E−14
INSRR_SDK1		153	6.3 E−33				
GFRA2_PTPRH		150	1.7 E−32				
PCDHB6_GFRA2		146	4.8 E−32				
INSRR_CDH24		142	1.7 E−31				
GPR173_GFRA2	7	140	2.4 E−31				
PTPRH_PCDHB6		134	1.3 E−30				
GPR173_PTPRH		130	4.6 E−30				
SDK1_PTPRH		130	2.0 E−30				
SDK1_GFRA2		128	8.1 E−30				
SDK1_GPR173		128	9.3 E−30				
CDH24_GFRA2	8	123	6.0 E−29				
SDK1_PCDHB6		111	2.0 E−27				
CDH24_PTPRH		106	1.1 E−26				
GPR173_PCDHB6		99	1.4 E−25				
CDH24_SDK1		97	2.6 E−25				
GPR173_CDH24	9	95	6.3 E−25				
CDH24_PCDHB6		81	1.8 E−22				

*Dual targets (hits) with favorable cell surface expression characteristics (Table 1) were ranked from the neuroblastoma without MYCN gene amplification^a^ or with MYCN gene amplification^d^ data sets according to MANOVA *F*-value^b^. Also listed is the MANOVA *p*-value^c^ for each pair. In gray boxes, the nine shared antigen pairs shared between both types are indicated*.

Of the 28 *MYCN*-A NB pairs, and the 45 *MYCN*-NA pairs listed in Table 2, nine are shared. As these common pairs (dyads) are upregulated together this may indicate that for both types of disease these cell surface proteins are reflective of a common cell of origin, or possibly a common disease process. ALK, GPCR173, GFRA2, GFRA3, and CDH24 are associated with each other in pairwise association 12 times in the top 25 dyads for *MYCN*-NA NB and 10 times for *MYCN*-A NB. The potential interaction of each of these molecules in RET signaling (except for ALK), indicates that this “pair” may be a linked activation complex in neuroblastoma. That these molecules are all involved in neuronal development and cellular response to migratory or chemotactic factors also is reflective of their neural crest origin. To further develop our bioinformatics analysis, we carried out pathological studies.

**Figure 2:**
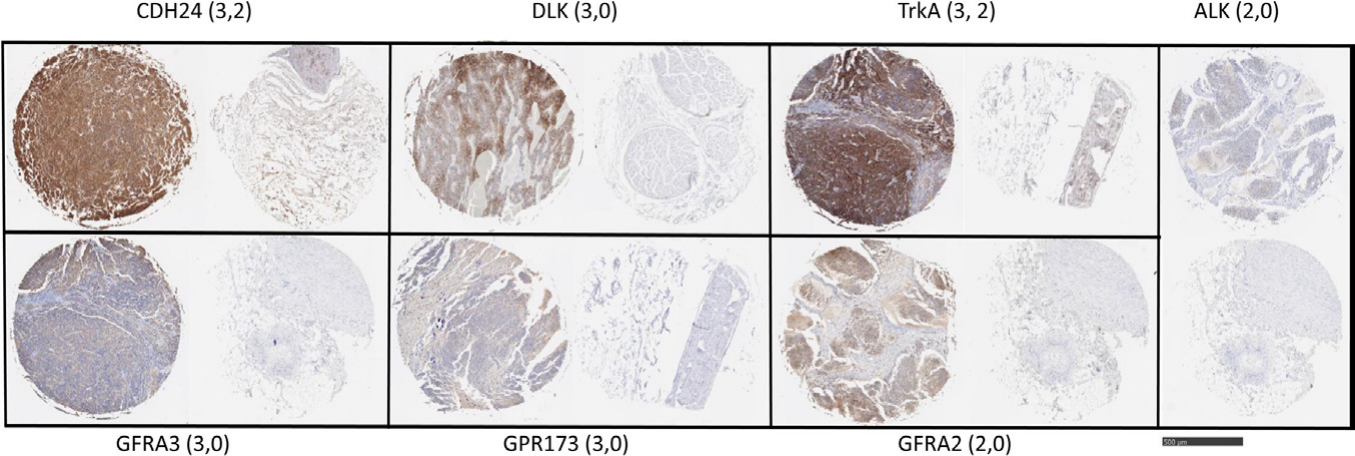
Target antigen expression in neuroblastoma and normal tissue arrays. Selected images from neuroblastoma tissue array were stained with the indicated antibodies. Shown are representative highest scores, in parentheses, for disease and normal (peripheral nerve) cores from the same staining array, respectively. Bar = 500 μm.

**Figure 3:**
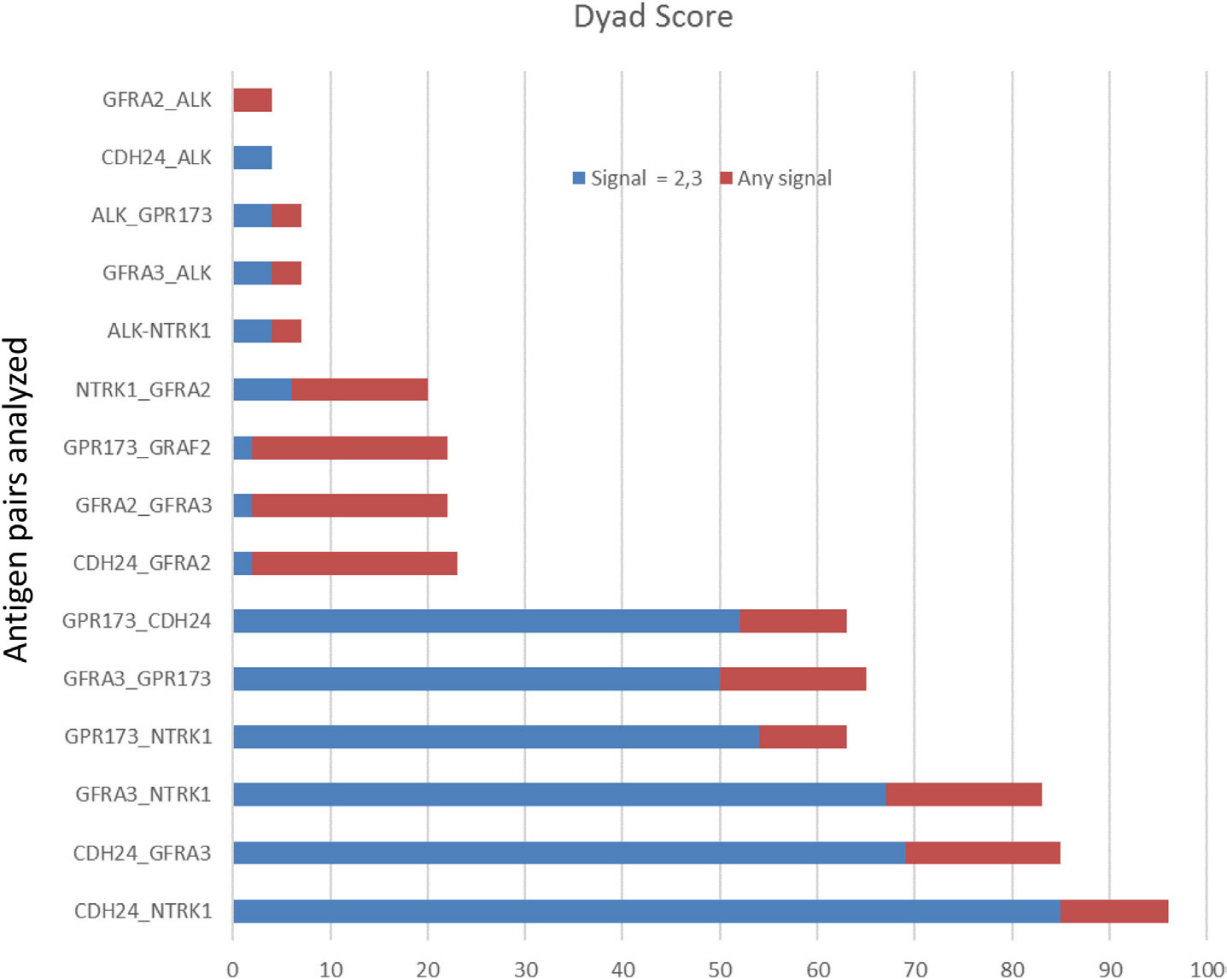
Target protein expression by staining intensity. Each core of the stained tissue arrays was scored as 0, 1, 2, or 3 for target protein expression. Dyad pairs identified by bioinformatics (*y*-axis) were placed in a scoring matrix wherein any signal above 0 (stacked red bar) or scored a 2 or 3 (blue stacked bar) was recorded and the percentage of biopsy scores expressing both antigens across each individually stained array counted. Data are presented as a percent of total specimens (*n* = 54) staining positive (Dyad score).

To examine protein expression, neuroblastoma tissue arrays were obtained and commercial antibodies that could be readily adapted to automated staining were used to probe the protein expression of ALK, DLK, NTRK1 (TrkA), GFRA2, GFRA3, GPR173, and CDH24, Figure 2. Each antibody was verified for binding activity and specificity by staining of normal tissue blocks prepared in the Department of Pathology at the Medical College of Wisconsin (see Materials and Methods). Each biopsy was then scored on a four-point scale (0, 1, 2, 3) for staining intensity. The results across all biopsies are summarized in Figure 3. A low-power image is provided to illustrate the scoring for 20 of the 54 individual cores, Figure S1 in Supplementary Material. Peripheral nerve was largely negative for ALK, DLK, GFRA2, GFRA3, GPR173, and somewhat positive for CDH24 and TrkA, Figure S2 in Supplementary Material. Staining of pathological tissues from in-house control blocks was used to confirm the staining activity of each antibody used in the neuroblastoma tissue arrays, Figure S3 in Supplementary Material. When the frequency of our bioinformatics hits for dual expression was explored in pathological sections, 6 dyads (pairwise over expressed antigens) were present in more than half of the array specimens tested. These were: (a) GPR173, GFRA3, or NTRK1 paired with CDH24, or (b) GFRA3 paired with NTRK1 or GPR173, or (c) GPR173 paired with NTRK1. These data illustrate the importance of verification of bioinformatics hits at the protein level. While we cannot conclude that these are a definitive picture of the neuroblastoma surfaceome, these data give us a place to start in designing new immunotherapeutic approaches to treat the disease.

## Discussion

**Figure 4:**
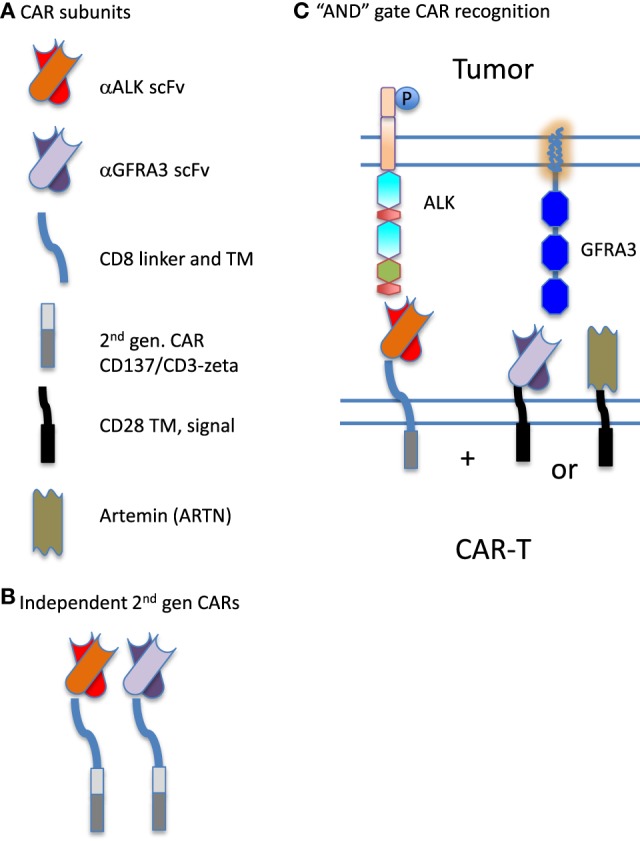
Synthetic biology approach to creating chimeric antigen receptor (CAR) “AND” gates. **(A)** CARs are composed of distinct combinatorial units, specifically: individual antigen-binding motifs [scFv specific for anaplastic lymphoma kinase (ALK) or GFRA3], linking domains (CD8 linker and TM) and intracellular signaling domains (illustrated are components of a second-generation CAR derived from CD137 and CD3-zeta, and an incomplete intracellular signaling domain composed of only CD28 transmembrane and signaling sequences). In addition to scFv, ligands for overexpressed receptors on the tumor surface such as Artemin, the ligand for GFRA3, can be used in CAR construction. **(B)** Two antigens on a tumor cell could be targeted by expressing two complete and independent CARs on the T cell surface, creating a logical “OR” gate for antitumor targeting. Either construct can fully activate the CAR-expressing lymphocyte (CAR-T) expressing it. **(C)** CAR components can also be used to split activation signals and thus require two binding interactions to fully activate the CAR-expressing lymphocyte, creating a logical “AND” gate. Here, the scFv specific for ALK expresses only one intracellular signaling domain. Complete activation of the T cell requires the binding of a second CAR. The second portion of the “AND” gate may be contributed by either an scFv specific for GFRA3 or the natural ligand of the tumor-expressed receptor, artemin (ARTN).

In previous work to define transcripts overexpressed in pediatric solid tumors, we were struck by the difficulty in finding antigens that are overexpressed on tumor cells but not on normal tissue, and thus able to serve as therapeutic targets. Moreover, when creating a tumor target antigen list based on differential expression between normal and tumor tissue, the final target list can change depending on which normal tissues are included in the analysis (26). In our current bioinformatic approach, using the latest RNASeq data for neuroblastoma, we filtered for both normal tissue in aggregate, and with a greater stringency filter for a set of tissues designated as essential. We included a filter for CNS expression alone, and then for a set of vital tissues (heart, lung, kidney, liver), Figure 1. This approach yields a gene set that differs from normal to a now measurable degree, but these hits are still not entirely unique for neuroblastoma. Given the need for safety using highly active cell-based immunotherapy, the investigator is faced with a need to be able to target the tumor surfaceome in a more sophisticated manner. One approach is to create a more complex CAR activation signal, for example, one that requires two specific “hits” for activation, Figure 4. The inverse could also be attempted by creating a CAR-T cell product that is negatively regulated by an antigen that is overexpressed on a vital normal tissue. For negative signals, both intracellular phosphatases such as CD45 and the intracellular signaling motifs of the checkpoint molecules PD-1 and CTLA-4 have been proposed (67, 68). Thus, a CAR specific for tumor-associated antigen “A,” to which an active intracellular signaling domain is spliced, will react to any cell that expresses “A,” unless antigen “B” is present, which is targeted by a second CAR expressed by the same T cell, yet to which inhibitory signaling domains are linked. “Split CAR” approaches have also been proposed, wherein binding to “A” delivers only a partial signal, and full activation to the point of target cell cytolysis requires a second binding event, “C,” that is also present on the tumor cell. This process can also be approached pharmacologically, wherein a CAR specific for “A” does not contain any T cell activation signals, but does contain a small-molecule-binding domain that can induce protein–protein association. In the same lentiviral vector, a second transcript, “D,” is expressed that would contain a complementary dimerization domain linked to the intracellular activation motifs. This small molecule dimerizer thus becomes an “on switch” and is required for full CAR activity. Chemical inducers of dimerization (CID domains) have been widely developed using the rapamycin/FK506 and FK506-binding protein system and have been recently reviewed by Wu et al. (69). However, none of these options has yet been put into clinical practice.

Although we set high filters for normal gene expression, it is apparent that the targets we have identified can be segregated into those with higher and lower risk, Table 1. Risky targets appear to reflect the neuroblastoma source tissue, which is of neural crest origin, and includes subunits of the nicotinic acetylcholine receptor (nAchR). nAchR is composed of five transmembrane proteins and serves as a primary ionotropic receptor for muscle contraction in the neuromuscular junction and is composed of different subunits in the CNS versus ganglia. Each member of the channel has four transmembrane domains with the N and C termini facing outwards into the extracellular space. The ability to target this receptor by the immune system is exemplified by myasthenia gravis, where antibodies block the ability of acetylcholine to bind to the receptor. On chromaffin cells the nAchR is formed by CHRNA3 and CHRNB4 (70), and these are the very receptors we find overexpressed in our gene expression analysis, attesting to the neural crest derivation of both chromaffin cells and neuroblastoma.

To develop a bioinformatic approach for advanced CAR-based therapy, we used gene expression data to identify targets overexpressed in tandem and scored as a pair against their expression in normal tissue. The identification of ALK was hardly surprising given its established association with neuroblastoma (61). Of interest is the discovery of GFRA2 and GFRA3 as overexpressed antigens as well. Both proteins signal in association with RET. In 2014, Cazes et al. reported that ALK triggers RET upregulation in mouse sympathetic ganglia at birth, and in human neuroblastoma (71). These authors proposed using a combination of crizotinib and vandetinib, inhibitors of ALK and RET, respectively, as a potential therapeutic approach. A phosphoproteomic analysis of neuroblastoma also found RET to be overexpressed and activated in neuroblastoma (72). Lambertz et al. recently described ALK-driven upregulation of MAPK regulators and RET in neuroblastoma, and also the RET-driven upregulation of cholinergic lineage markers (73). Once again, combined blockade of the ALK and RET pathways was proposed. Serial analysis of gene expression-analysis in a neuroblastoma cell line revealed another of our identified targets, DLK-1, to be highly upregulated. The involvement of the delta-notch signaling pathway in neuroblastoma has also been described (74). Interestingly, the combination of retinoic acid and the knockdown of DLK1 was found to induce the differentiation of neuroblastoma cells *in vitro* better than either intervention on its own, highlighting the tumorigenicity of DLK1 in neuroblastoma (75).

The targets identified in our tandem gene discovery process appear to be valid targets in their own right. However, using strategies that require two different antigens on the tumor cell surface to activate CAR-T adds an added layer of safety and specificity, and may open the door to new therapeutic approaches for other solid cancers. Another important finding of our study is that many of the cell surface proteins identified, comprising the neuroblastoma surfaceome, encode functional receptors. The ligands for these receptors can also be used as loci of CAR-T activation, as has been described for the IL-13-based zetakine approach (76) (Figure 4). As with any RNA-based bioinformatic approach, our findings will require extensive confirmation with protein-detection based analysis. Histochemical analysis of neuroblastoma tissue cores revealed that only a few of our identified tandem antigenic pairs were expressed on the majority of specimens. The lack of strong ALK and GFRA2 expression at the protein level highlights the need to validate analysis at the RNA level with appropriate proteomic characterization of the tumor type being studied. The proteins pairs identified in Figure 3 as having high expression by antigen staining (CDH24, NTRK1, GFRA3, and GPR173) will now serve as the starting point for the development of bispecific CAR-T approaches to neuroblastoma therapy. Our plan is to develop CARs that functions as a logical “and” gates, requiring the successful engagement of two binding moieties to initiate immune effector cell function.

## Author Contributions

Studies were planned and designed by RO and JK, bioinformatic pipelines, and statistical analysis of gene expression was performed by SS, XW, JH, and JW. Pathology studies were designed and carried out by JJ and CD, JJ also responsible for normal tissue blocks, automated processing, and scoring. Final data were analyzed by RO and JK, who also wrote the manuscript.

## Conflict of Interest Statement

RO is a full-time employee of Lentigen Technology, Inc., a Miltenyi Biotec Company. All other Authors declare that the research was conducted in the absence of any commercial or financial relationships that could be construed as a potential conflict of interest.

## Supplementary Material

Figure S1 Low power images of neuroblastoma tissue cores. Twenty out of a total of 54 cores are shown. Each core is from retroperitoneal disease with the exception of the two most rightward cores on the second (lower) row, which were obtained from mediastinal disease. Each panel shows immunohistochemical staining for anaplastic lymphoma kinase, CDH24, DLK, GFRA2, GFRA3, GPR173, and TRKA as indicated above each panel. In the lower right of each core is the score assigned by pathological examination.

Figure S2 Low power images of peripheral nerve and high power images for TrkA staining. Six of the 10 normal peripheral nerve tissue cores used as negative control are shown. As listed on the left, each of the six cores was stained with the indicated antibody. Staining intensity score is indicated in the lower right corner of each section. In some cores, no score could be determined due to loss of tissue (ND). In the final two panels, high power images illustrate the difference between moderately strong (score of 2) and negative (score of 0) tissue. Bars indicate magnification for low power, 5 mm, and high power, 100 μm, respectively.

Figure S3 Control tissue block staining to validate staining. As described in Section “Materials and Methods,” (1) anaplastic lymphoma kinase-1-stained control tissue blocks with cerebellum, pancreas, tonsil, and lymphoma (ALCL) were utilized (CBlock1). Representative fields for ALCL (1a, strong positive) and tonsil (1b, negative) are shown. In all figures, the solid bar denotes scale. Staining from the same block shows a bar for the first field only (unless a higher power image is shown). (2) CDH24 was tested on CBlock1 with added bowel, uterus, cerebellum, and HCC. Ganglion cells/nerve cells of the bowel stained positive, endometrium surface epithelium cytoplasmic positive, and HCC was strongly positive. ALCL (2a), tonsil (2b), and uterus with strong staining of the basal layer (2c) are shown. (3) DLK1 was tested on CBlock1 with added pancreas and placenta. ALCL (3a), tonsil (3b), and placenta (3c, strong positive) are shown. Islets, ductal epithelium, and neutrophils also stained positive. (4) GFRA2 was tested on CBlock1 with added lung and liver. Staining was positive for macrophages and sinusoids in liver, tonsil leukocytes (4b), ALCL was weakly positive (4a), and lung unremarkable. (5) GFRA3 was tested on CBlock1 with added lung and liver. Increased expression in pancreatic ductal epithelium and islets, strong lymphatic vessel staining, and positive alveolar macrophages were seen. ALCL (5a) tonsil (5b), and HCC (5c) are shown. (6) GPR173 was tested on CBlock1 with added lung, liver, and skin. Positive staining was seen on red cells and lymphocytes. ALCL (6a), tonsil (6b), and HCC (6c) are shown. (7) TrkA was tested on CBlock1 and liver (7a). Positive staining was seen in tonsil reticular dendritic network (7b), pancreas islets, sinusoids of HCC, and some hepatocytes were positive, as was liver artery (7c).
