# Supplementary figures and images for: Paired Expression Analysis of Tumor Cell Surface Antigens
Source: Front Oncol. 2017 Aug 21;7:173. doi: 10.3389/fonc.2017.00173 (PMC5566986; doi:10.3389/fonc.2017.00173)

ALK

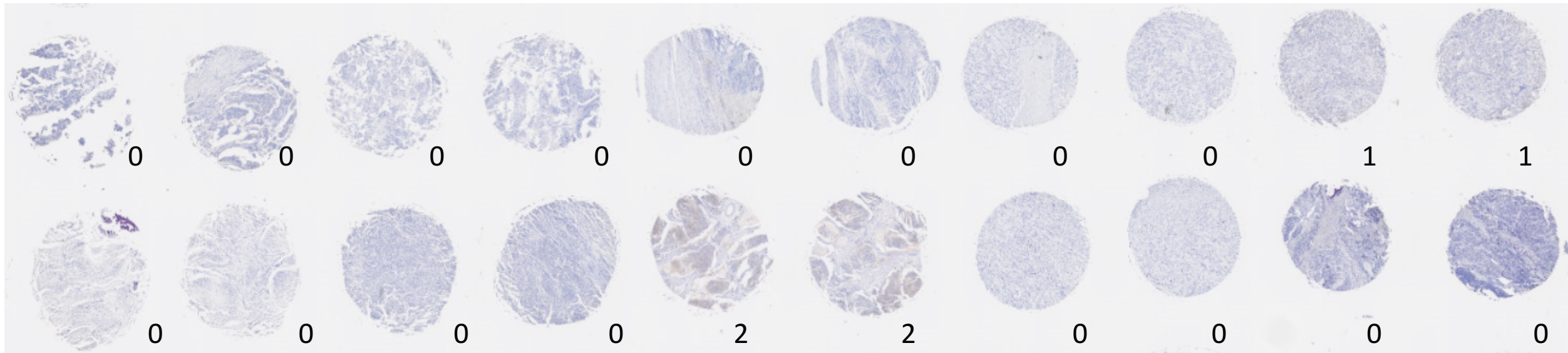

CDH24

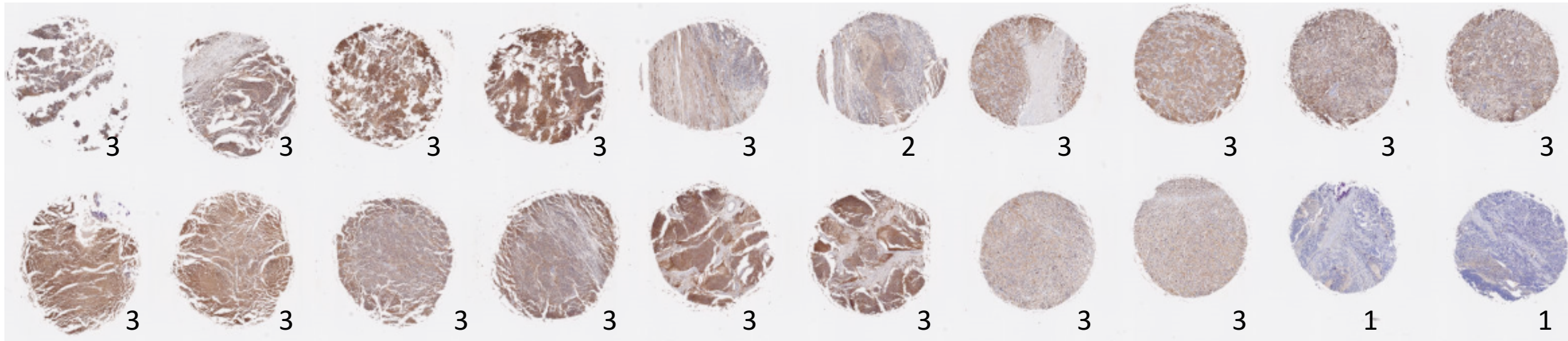

DLK

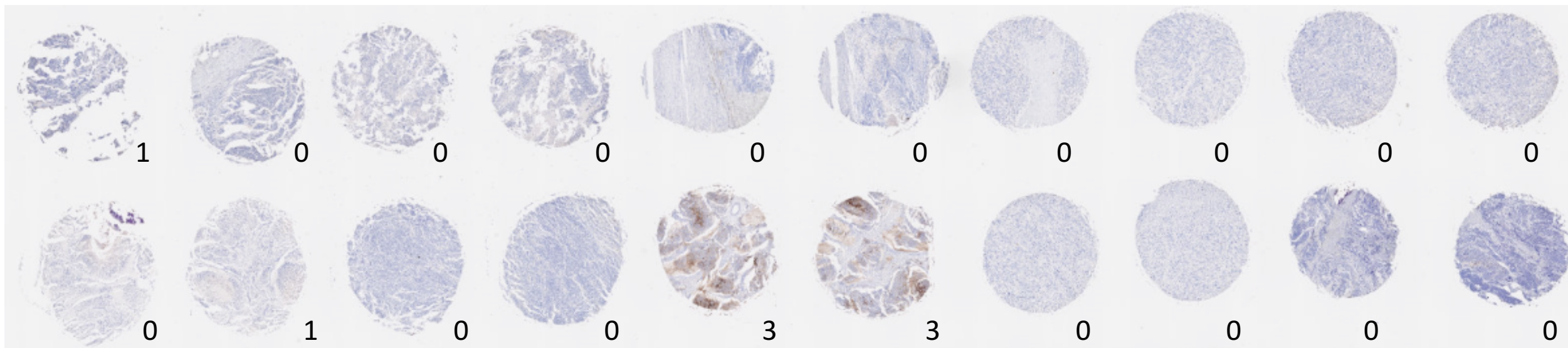

GFRA2

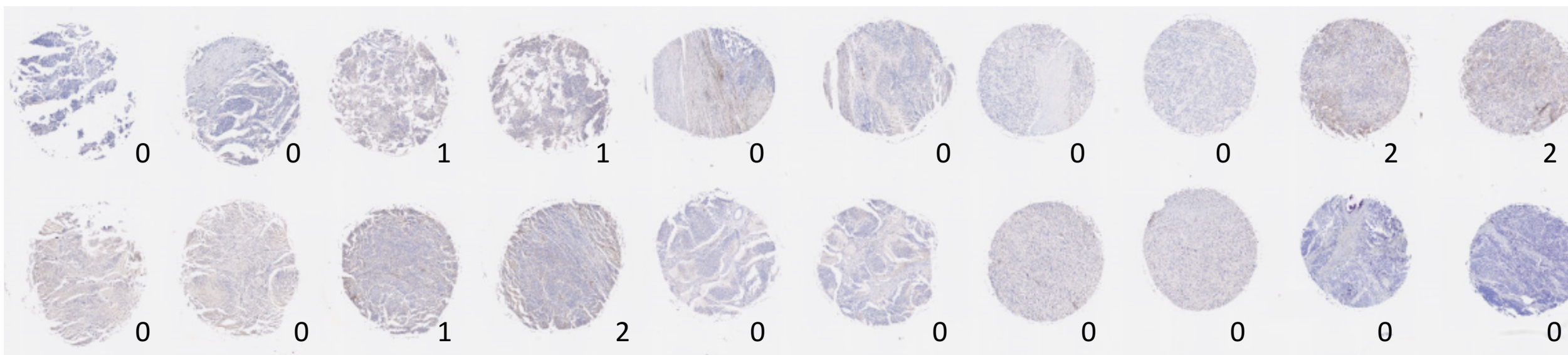

GFRA3

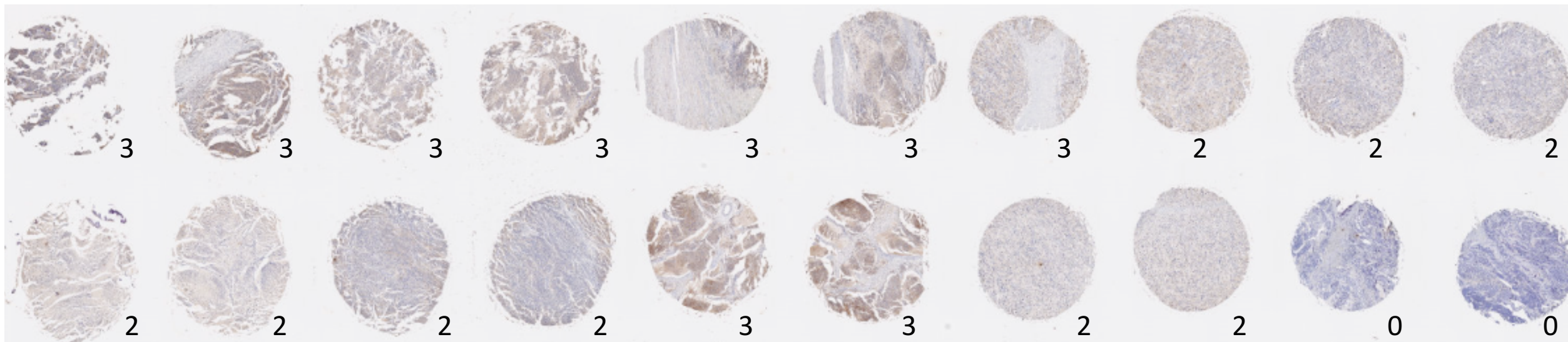

GPR173

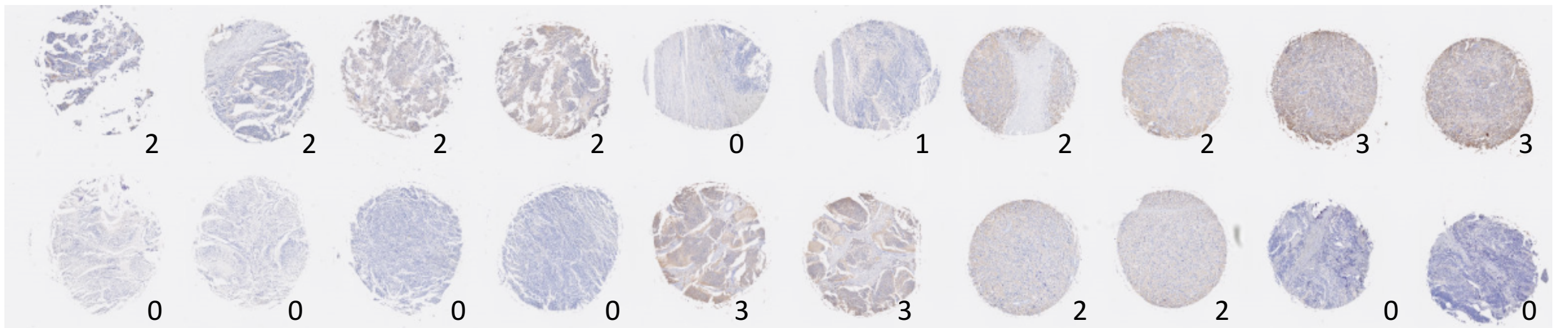

TRKA

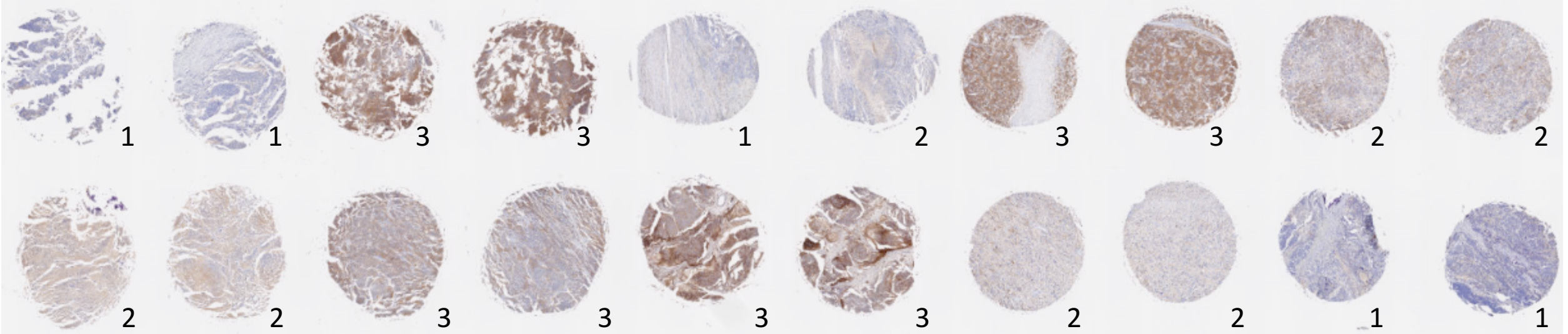

Supplement: Figure S1 — Low power images of neuroblastoma tissue cores. Twenty out of a total of 54 cores are shown. Each core is from retroperitoneal disease with the exception of the two most rightward cores on the second (lower) row, which were obtained from mediastinal disease. Each panel shows immunohistochemical staining for anaplastic lymphoma kinase, CDH24, DLK, GFRA2, GFRA3, GPR173, and TRKA as indicated above each panel. In the lower right of each core is the score assigned by pathological examination. [file Image_1.PDF]

ALK

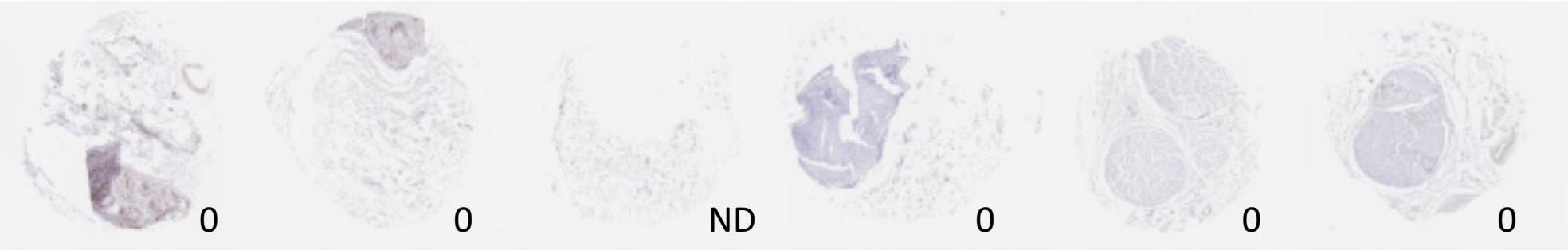

CDH24

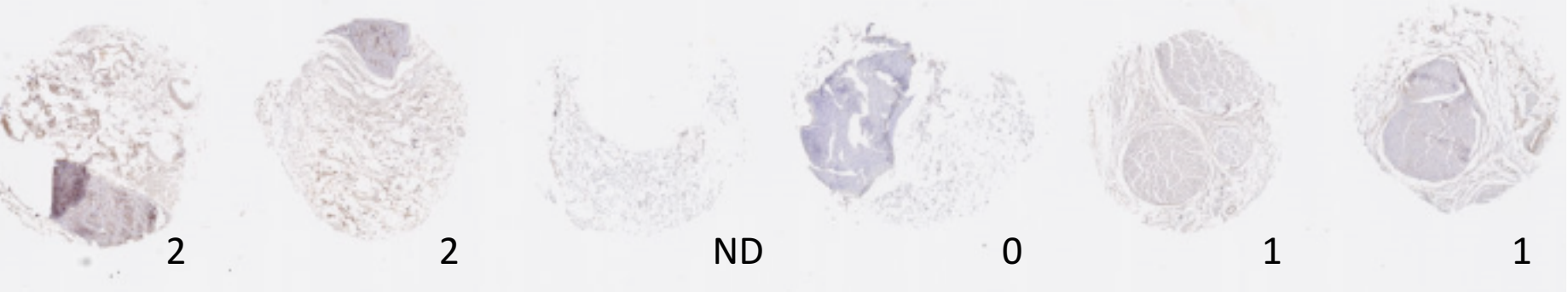

DLK

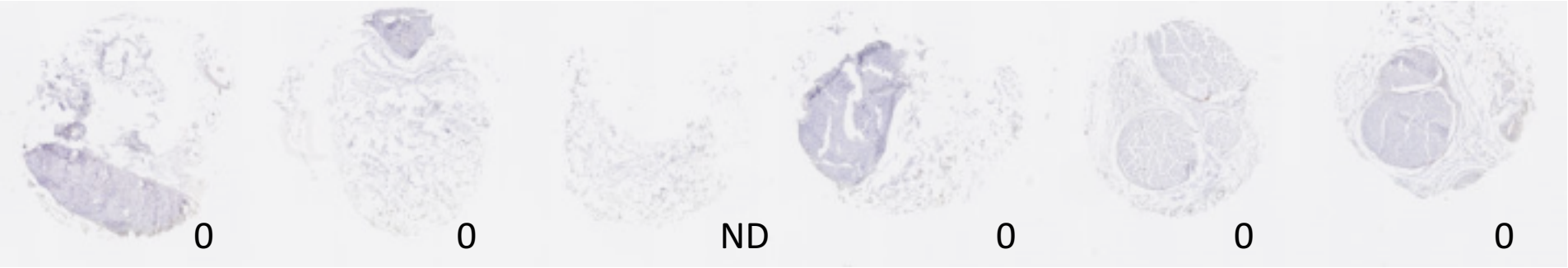

GFRA2

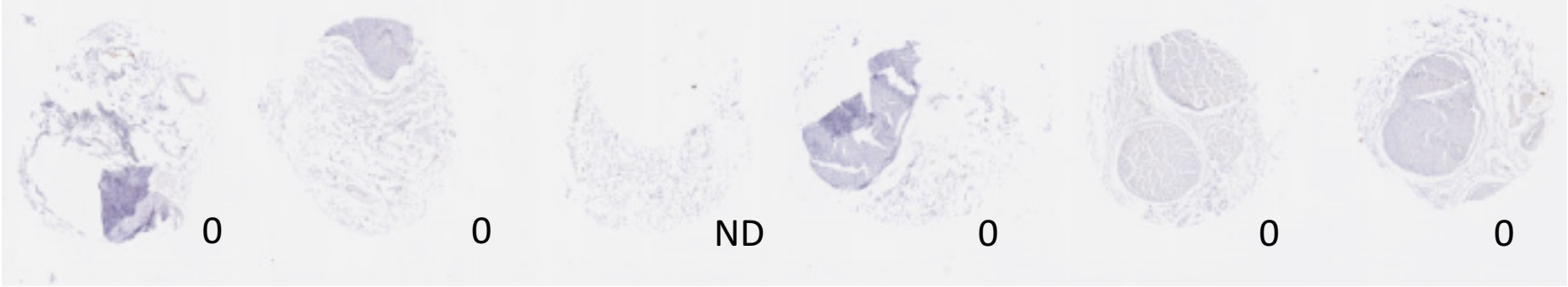

GFRA3

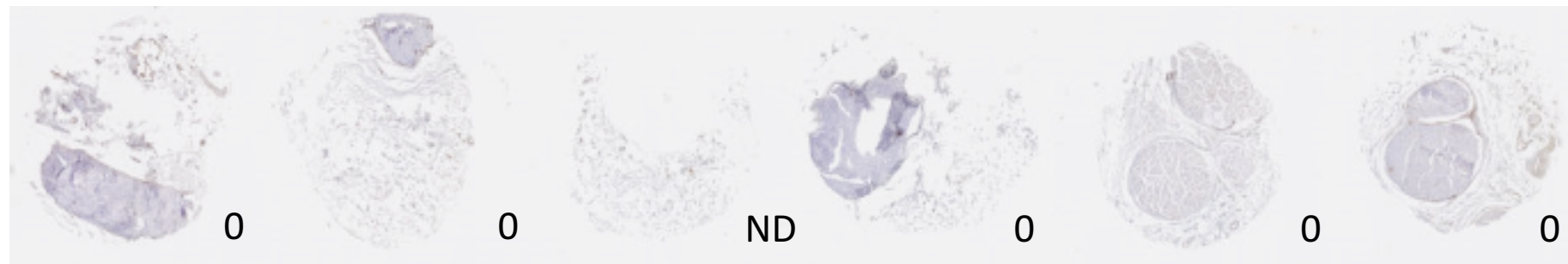

GPR173

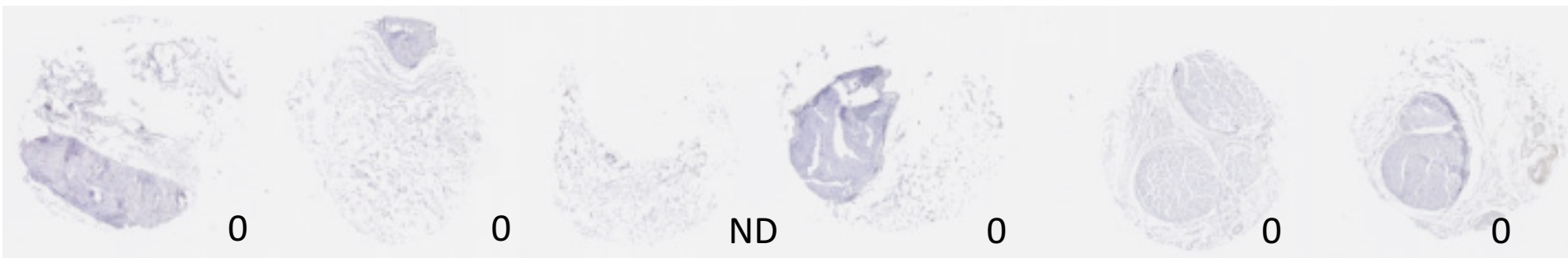

TRKA

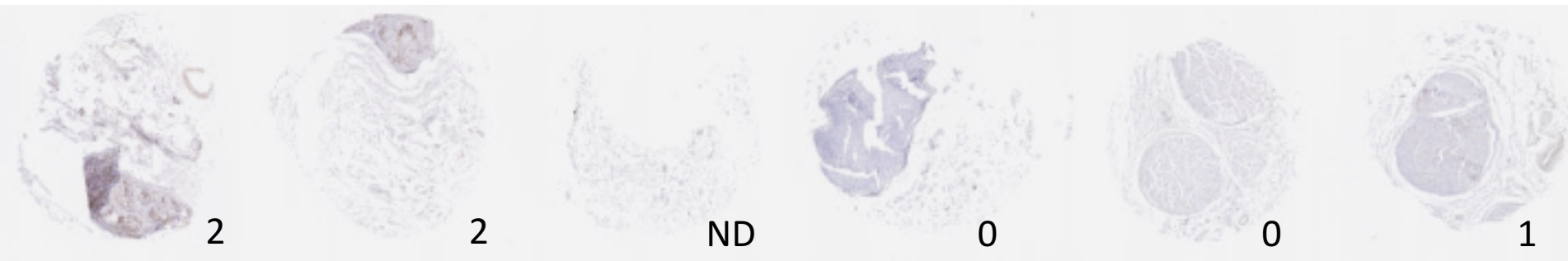

5 mm

TRKA (2)

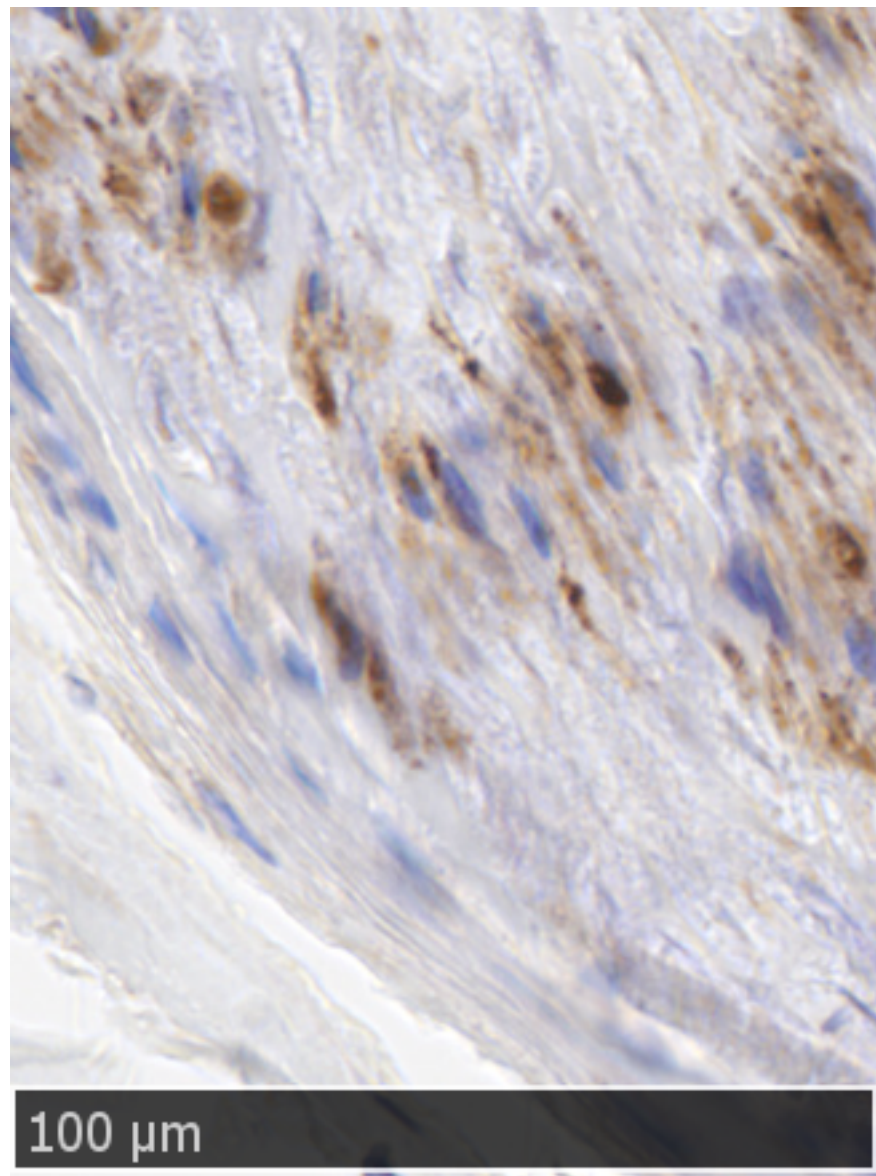

TRKA (0)

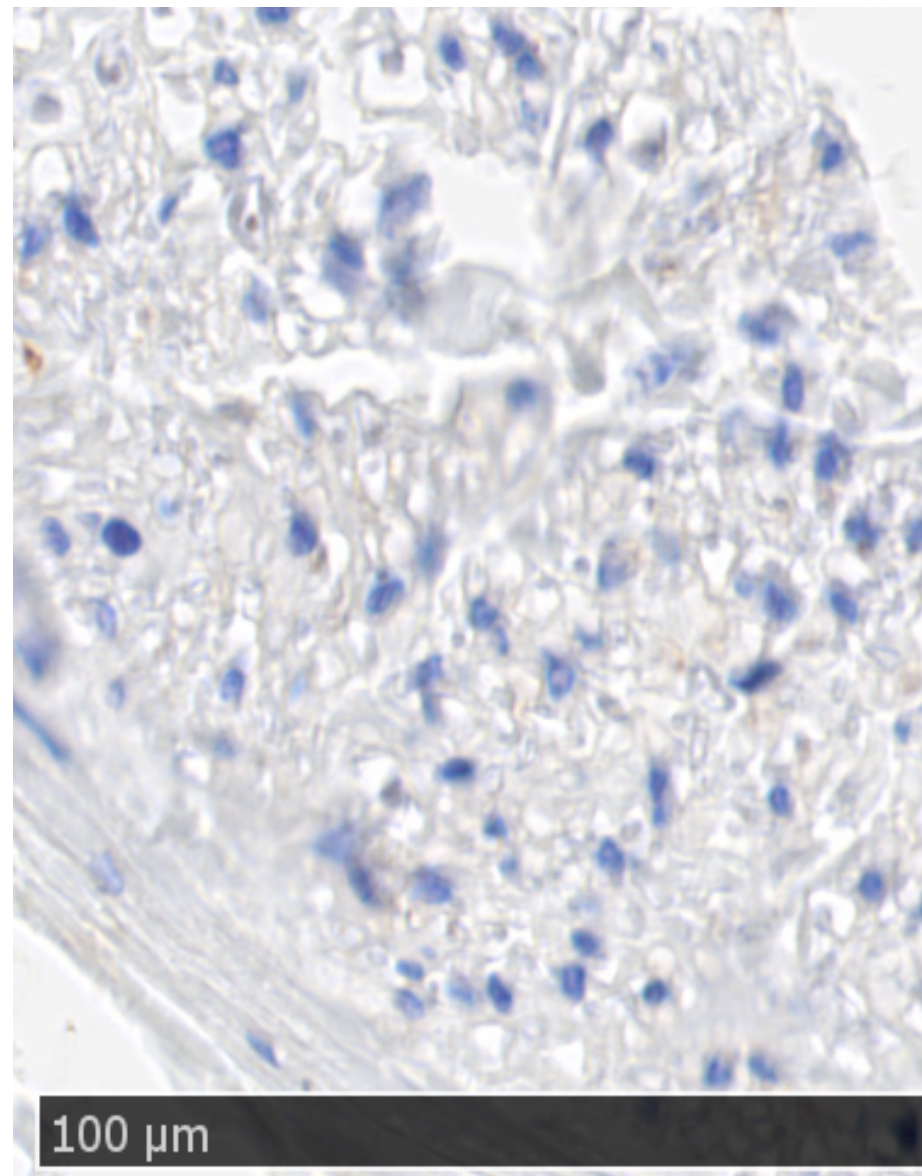

Supplement: Figure S2 — Low power images of peripheral nerve and high power images for TrkA staining. Six of the 10 normal peripheral nerve tissue cores used as negative control are shown. As listed on the left, each of the six cores was stained with the indicated antibody. Staining intensity score is indicated in the lower right corner of each section. In some cores, no score could be determined due to loss of tissue (ND). In the final two panels, high power images illustrate the difference between moderately strong (score of 2) and negative (score of 0) tissue. Bars indicate magnification for low power, 5 mm, and high power, 100 μm, respectively. [file Image_2.PDF]

1. ALK

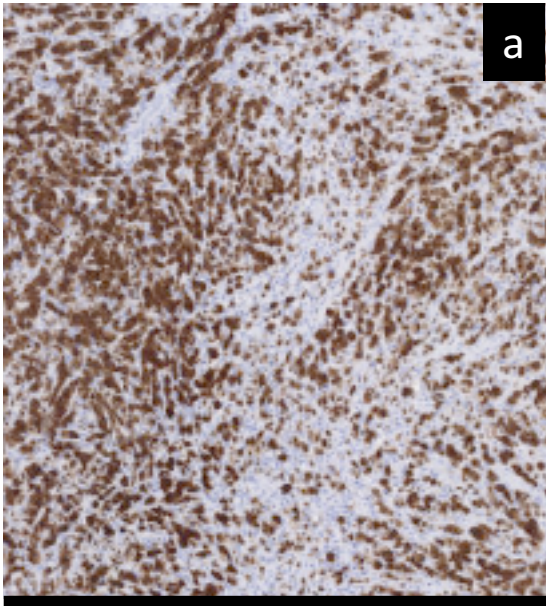

a

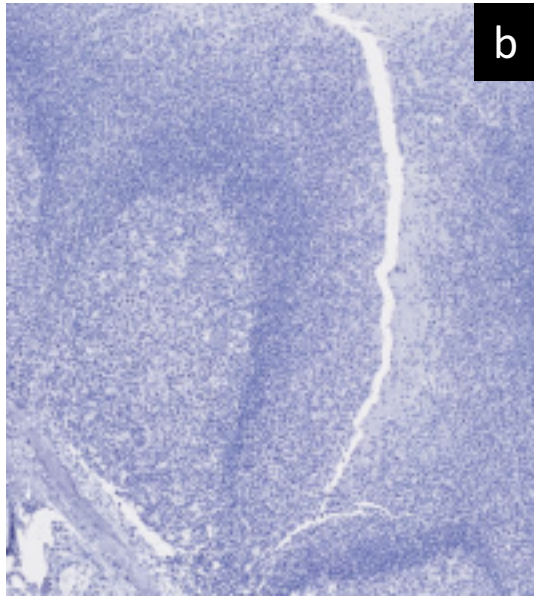

b

1 mm

2. CDH24

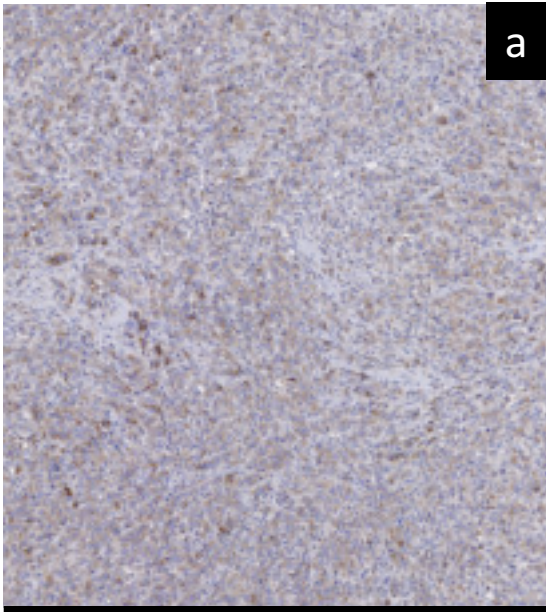

a

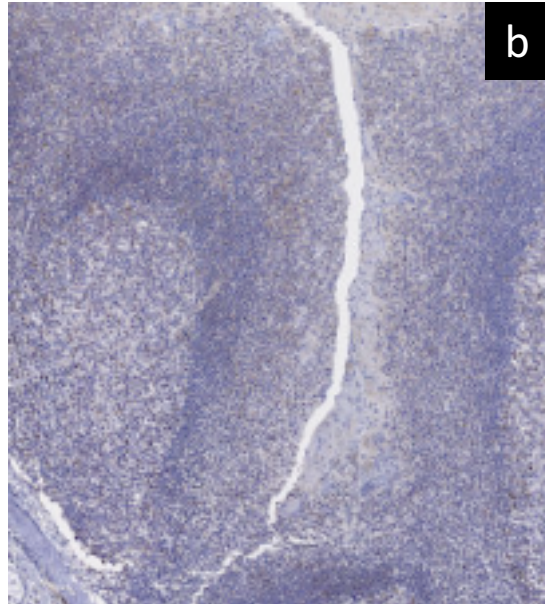

b

1 mm

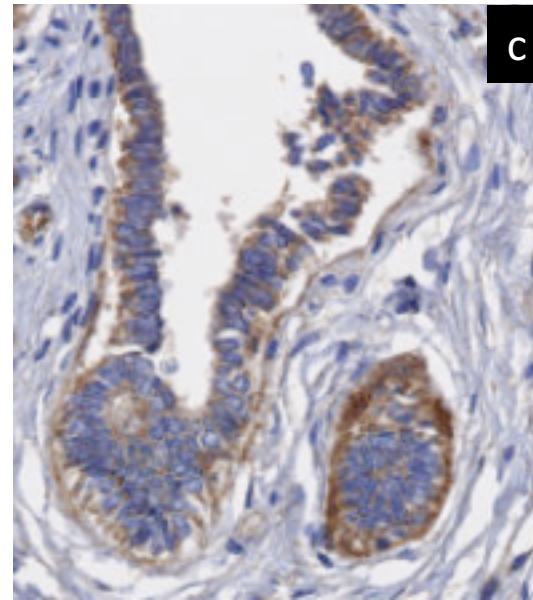

c

250 um

### 3. DLK

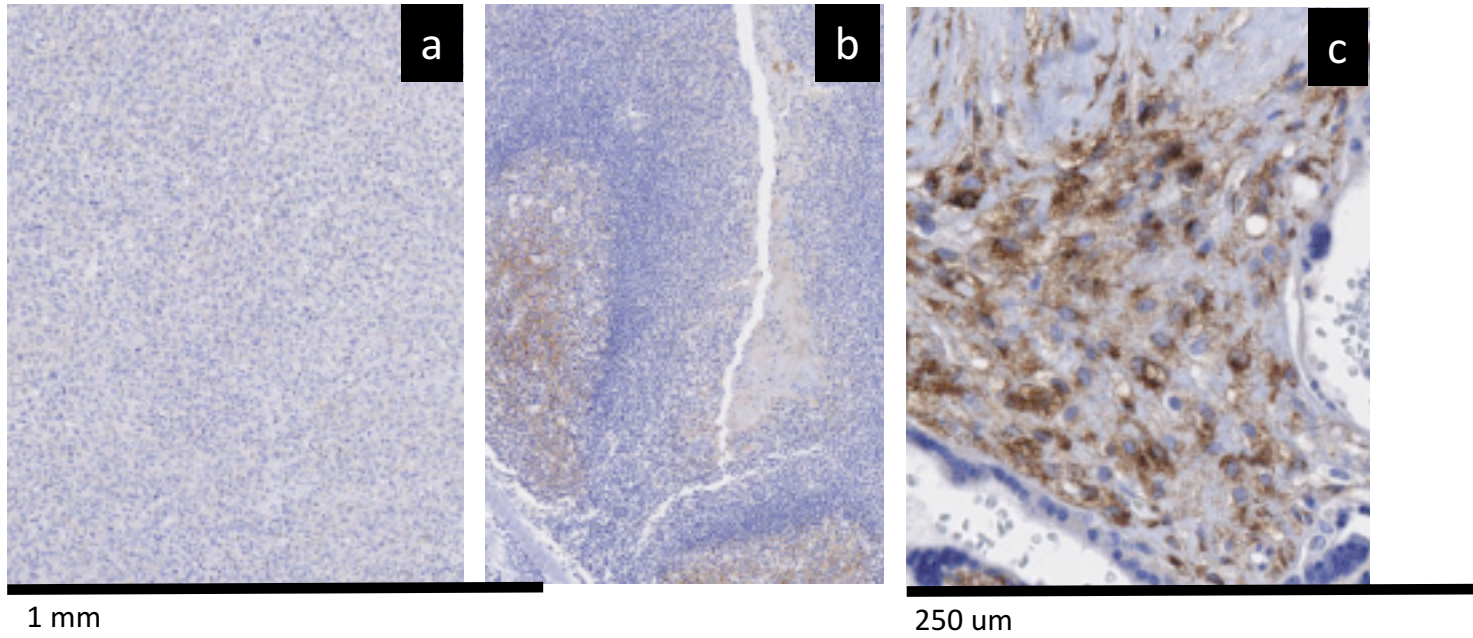

### 4. GFRA2

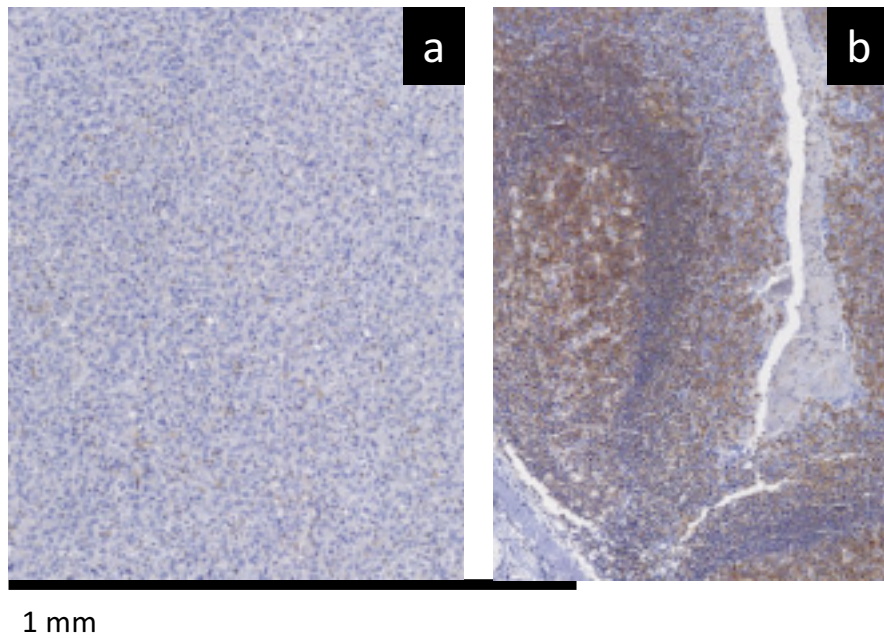

5. GFRA3

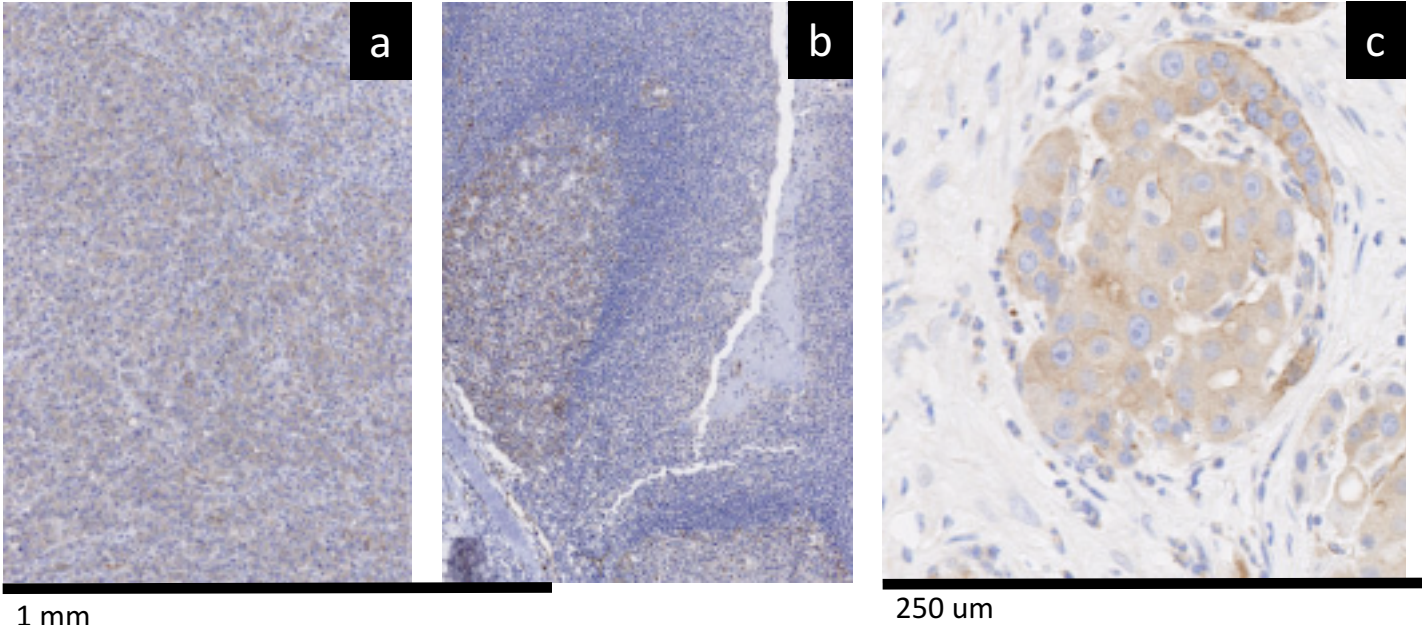

6. GPR173

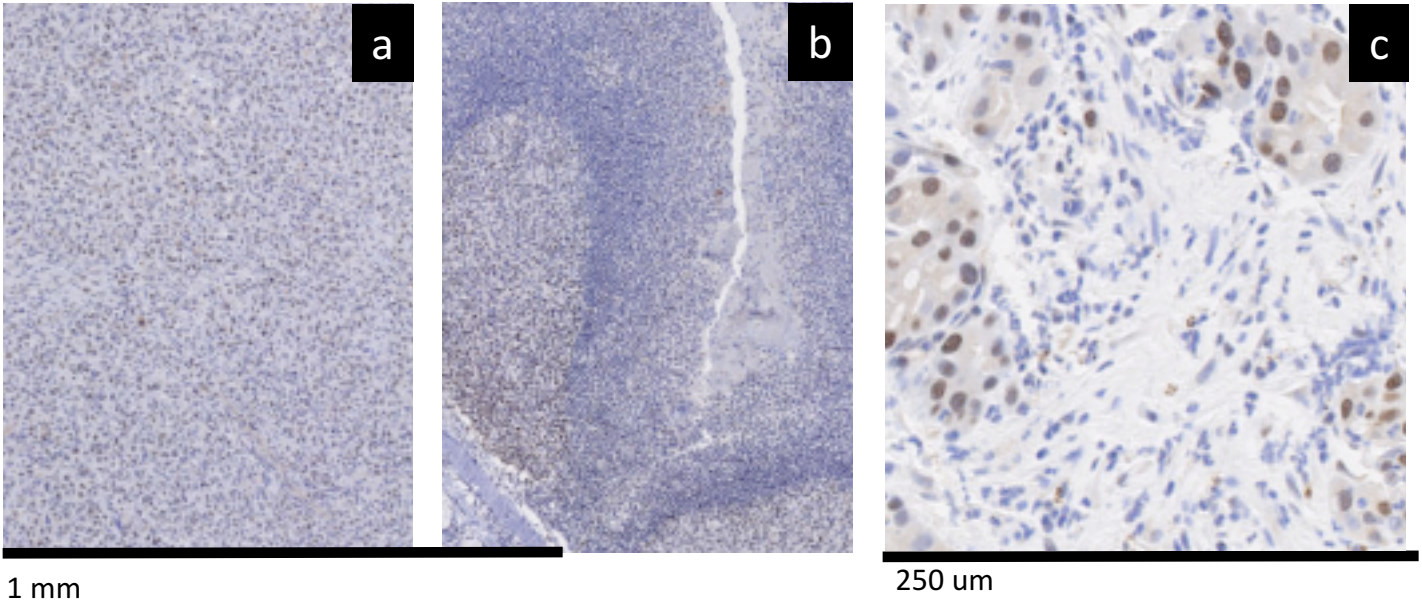

## 7. TRKA

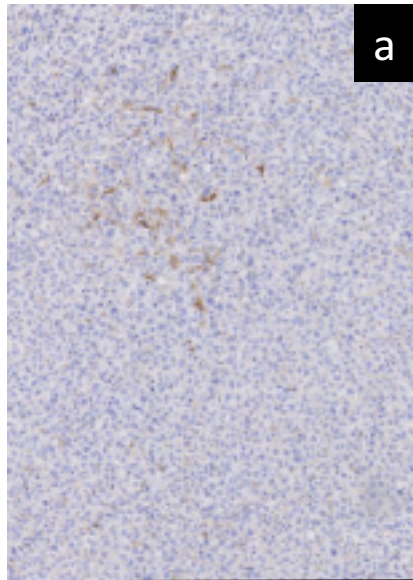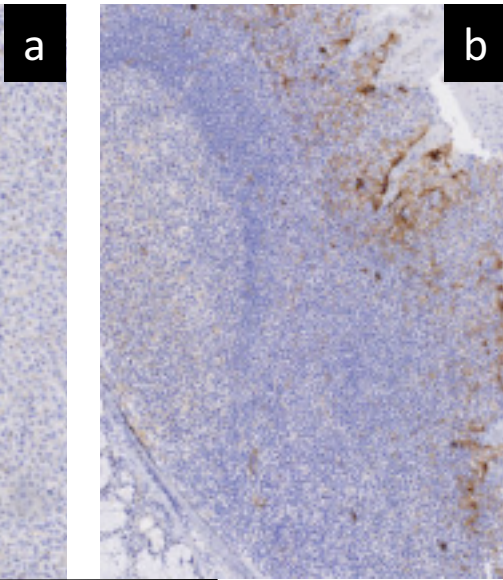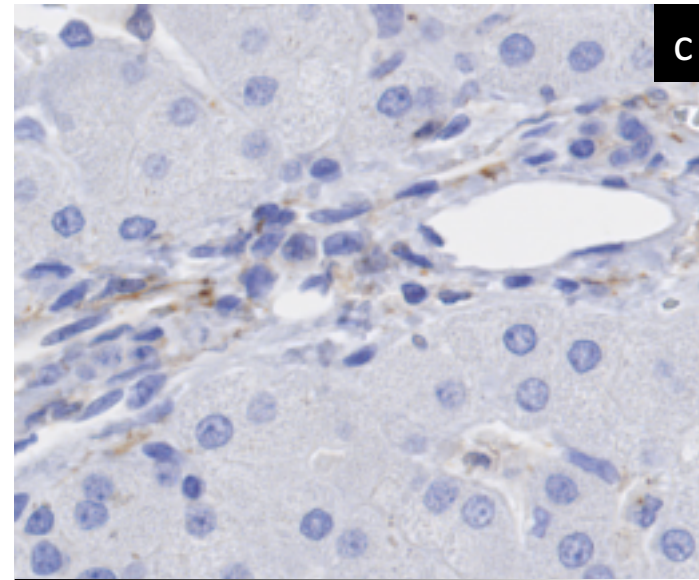

Supplement: Figure S3 — Control tissue block staining to validate staining. As described in Section “Materials and Methods,” (1) anaplastic lymphoma kinase-1-stained control tissue blocks with cerebellum, pancreas, tonsil, and lymphoma (ALCL) were utilized (CBlock1). Representative fields for ALCL (1a, strong positive) and tonsil (1b, negative) are shown. In all figures, the solid bar denotes scale. Staining from the same block shows a bar for the first field only (unless a higher power image is shown). (2) CDH24 was tested on CBlock1 with added bowel, uterus, cerebellum, and HCC. Ganglion cells/nerve cells of the bowel stained positive, endometrium surface epithelium cytoplasmic positive, and HCC was strongly positive. ALCL (2a), tonsil (2b), and uterus with strong staining of the basal layer (2c) are shown. (3) DLK1 was tested on CBlock1 with added pancreas and placenta. ALCL (3a), tonsil (3b), and placenta (3c, strong positive) are shown. Islets, ductal epithelium, and neutrophils also stained positive. (4) GFRA2 was tested on CBlock1 with added lung and liver. Staining was positive for macrophages and sinusoids in liver, tonsil leukocytes (4b), ALCL was weakly positive (4a), and lung unremarkable. (5) GFRA3 was tested on CBlock1 with added lung and liver. Increased expression in pancreatic ductal epithelium and islets, strong lymphatic vessel staining, and positive alveolar macrophages were seen. ALCL (5a) tonsil (5b), and HCC (5c) are shown. (6) GPR173 was tested on CBlock1 with added lung, liver, and skin. Positive staining was seen on red cells and lymphocytes. ALCL (6a), tonsil (6b), and HCC (6c) are shown. (7) TrkA was tested on CBlock1 and liver (7a). Positive staining was seen in tonsil reticular dendritic network (7b), pancreas islets, sinusoids of HCC, and some hepatocytes were positive, as was liver artery (7c). [file Image_3.PDF]
